# ZBED6 Knockout Prevents Ageing‐ and Dexamethasone‐Induced Muscle Atrophy via Dkk3 in Pig and Mice

**DOI:** 10.1002/jcsm.13829

**Published:** 2025-06-04

**Authors:** Chengjie Wei, Dandan Wang, Yitian Ma, Shengnan Wang, Dengke Pan, Yuehui Ma, Lin Jiang

**Affiliations:** ^1^ Laboratory of Animal (Poultry) Genetics Breeding and Reproduction, Ministry of Agriculture, Institute of Animal Science Chinese Academy of Agricultural Sciences (CAAS) Beijing China; ^2^ National Germplasm Center of Domestic Animal Resources, Ministry of Science and Technology of the People's Republic of China, Institute of Animal Science Chinese Academy of Agricultural Sciences (CAAS) Beijing China; ^3^ Institute of Organ Transplantation Sichuan Academy of Medical Sciences & Sichuan Provincial People's Hospital Chengdu China

**Keywords:** ageing, dexamethasone, Dkk3, skeletal muscle atrophy, ZBED6

## Abstract

**Background:**

Effective treatments for skeletal muscle atrophy, a debilitating condition linked to ageing and glucocorticoid therapy, remain lacking. Zinc finger BED‐type containing 6 (ZBED6), a transcriptional repressor, enhances muscle growth and protects against sepsis‐induced atrophy, but its role in ageing‐ and dexamethasone (Dex)‐induced muscle atrophy remains unknown. This study investigated the protective role of ZBED6 knockout (KO) against muscle atrophy through the Dkk3‐Fbxo32 pathway.

**Methods:**

The muscle mass, ratio and myofibrillar morphology of 5‐day‐old (wild‐type (WT): KO, *n* = 5:3), 5‐month‐old (*n* = 8:9) and 8‐month‐old (*n* = 3:3) ZBED6‐KO pigs and 18‐month‐old mice (*n* = 3:3) were analysed. A model of Dex‐induced muscle atrophy was established using 3‐month‐old mice (*n* = 6:6) via intraperitoneal injections (15 mg/kg/day for 10 days). C2C12 myotubes were treated with 100 μM Dex for 24 h. Muscle morphology was analysed through H&E and immunofluorescence staining. Gene expression was assessed through RNA‐seq, qRT‐PCR and western blotting. The downstream targets were identified through ChIP‐seq using anti‐ZBED6 antibodies and RNA‐seq analysis of the gastrocnemius muscle from ZBED6‐KO and WT pigs. Dkk3 was overexpressed by injecting AAV9‐myo2A‐Dkk3 (2 × 10^11^) into the tibialis anterior muscle of 3‐month‐old ZBED6‐KO mice (*n* = 4), which were harvested 1 month postinjection. ZBED6‐KO C2C12 cells were generated via CRISPR/Cas9 and treated with Dex to assess the effects on myotube diameter and gene expression.

**Results:**

The muscle mass and muscle‐to‐carcass ratio in ZBED6‐KO pigs increased by 27% and 12%, respectively (*p* < 0.05), while the Dkk3‐Fbxo32 pathway was suppressed by 50% (*p* < 0.01). ChIP‐seq/RNA‐seq identified Dkk3 as the most significant ZBED6 target (log2FC = −3.38, *p* < 0.01). The myofibrillar cross‐sectional areas (CSAs) increased twofold in aged ZBED6‐KO mice, while fibrosis and the Dkk3‐Fbxo32 pathway were suppressed by 76% and 50%, respectively (all *p* < 0.01). Dkk3 overexpression reduced the tibialis anterior muscle weight and CSA in ZBED6‐KO mice by 31% and 61%, respectively (*p* < 0.01). Dex reduced the CSA in WT mice (45%, *p* < 0.01), but ZBED6‐KO mice resisted atrophy (CSA similar to untreated WT). ZBED6‐KO increased myotube diameter by twofold (*p* < 0.01) and inhibited the activation of the Dkk3‐Fbxo32 pathway (*p* < 0.01). Conversely, *Zbed6* overexpression reduced the CSA and myotube diameter by 32% and 64%, respectively (*p* < 0.01) and rescued by Dkk3 silencing (50% recovery, *p* < 0.01).

**Conclusions:**

ZBED6 depletion mitigates ageing‐ and Dex‐induced muscle atrophy via the Dkk3‐Fbxo32 axis, highlighting its therapeutic potential.

## 1 Introduction

Muscle atrophy diminishes the quality of life and can lead to disability and even death in severe cases. Muscle atrophy can exist in several forms, including sarcopenia, which is characterised by the loss of muscle mass and function in the elderly. Muscular dystrophy, another form of muscle atrophy, is a progressive condition characterised by muscle weakness and atrophy resulting from genetic disorders. Generalised muscle atrophy refers to the reduction in muscle size or mass, typically due to ageing, malnutrition or physical inactivity [1]. Muscle atrophy results from the gradual loss of muscle mass and function under chronic stress, which can be caused by various factors, including ageing, diabetes, obesity, sepsis and long‐term infections. Additionally, agonists of the glucose‐dependent insulinotropic polypeptide (GIP) receptor and other drugs used for the treatment of obesity can induce the loss of muscle mass [2]. These conditions and pharmacological agents increase the serum glucocorticoid levels to promote the net loss of proteins, organelles and cytoplasm from myofibres, all of which ultimately reduce myofibre volume [3]. Additionally, the age‐related loss of muscle mass is attributed to atrophy and a significant reduction in the CSA of myofibres [4].

The transcriptional activation of E3 ubiquitin ligases, such as Atrogin‐1/MAFbx (muscle atrophy F‐box protein; *Fbxo32*) and muscle RING finger‐1 protein (*Murf1*), plays a critical role in various forms of muscle atrophy [5, 6]. Fbxo32 contributes to the rapid loss of muscle mass, and the depletion of *Murf1* aids in the preservation of muscle mass during ageing [7]. Recent studies have demonstrated that the activation of *Dkk3* exacerbates age‐related muscle atrophy by promoting the recruitment of β‐catenin to the binding sites of Forkhead box O3 (*FoxO3*), a member of the Fox family of transcription factors, located in the promoter regions of genes encoding Fbxo32 and Murf1 [8]. Additionally, the elevation in the levels of glucocorticoids promotes their binding to the glucocorticoid receptor (GR), which subsequently induces the nuclear translocation and recruitment of GR to the glucocorticoid response element (GRE). This process is also essential for the transcriptional activation of *FoxO3*, which in turn activates the transcription of *Fbxo32* and *Murf1* [9, 10, 11], to promote the glucocorticoid‐induced degradation of muscle proteins. Despite extensive studies, there are no identified therapeutic targets for the treatment of skeletal muscle atrophy induced by ageing or elevated glucocorticoid levels.

The zinc finger BED‐type containing 6 (*ZBED6*) protein is a transcriptional modulator that is unique to placental mammals. Studies on pigs and mice have demonstrated that the depletion of *ZBED6* promotes muscle growth and increases cardiac muscle volume by targeting intron 3 of *IGF2* [12, 13, 14]. Our recent studies on pigs indicate that ZBED6 modulates muscle mass by targeting other genes, including cyclin‐dependent kinase inhibitor 1A (CDKN1A) and dedicator of cytokinesis 3 (DOCK3) [15]. The loss of ZBED6 upregulates the expression of DOCK3, leading to the hyperactivation of its downstream RAC1/PI3K/AKT signalling cascade, which in turn attenuates sepsis‐induced muscle atrophy [16]. In this study, we demonstrated that the depletion of *ZBED6* markedly increased the CSA of the myofibres in adult pigs and aged mice. By integrating ChIP‐seq and RNA‐seq analyses, we identified that *Dkk3* is a direct positive target of *ZBED6* and observed that *ZBED6* knockout (ZBED6‐KO) downregulated the transcription of *Dkk3*. The findings additionally demonstrated that Dex, a glucocorticoid analogue, induced myotube atrophy and significantly upregulated the expression of *Zbed6* in C2C12 cells, suggesting a novel role for Zbed6 in Dex‐induced muscular atrophy. The results of in vitro and in vivo experiments confirmed that the ZBED6‐Dkk3 axis plays a critical role in the FoxO3/Fbxo32/Murf1 pathway to modulate the muscular atrophy induced by ageing or glucocorticoids (Figure S1). The findings further revealed that the Zbed6‐Dkk3 axis can serve as a promising therapeutic target for preventing muscle atrophy induced by ageing and glucocorticoids.

## 2 Methods

### 2.1 Animals and Treatment

The animal experiments were performed in accordance with the regulations and guidelines established by the Animal Care Committee of the Beijing Academy of Agricultural Sciences (approval number: IAS2022‐144).

ZBED6‐null Bama miniature pigs were generated as previously described [15], and the animals were bred at a pig farm affiliated with the Institute of Animal Science, Chinese Academy of Agricultural Sciences (CAAS), Beijing, China. The Zbed6‐null mice were provided by Leif Andersson from the Science for Life Laboratory at the Department of Medical Biochemistry and Microbiology, Uppsala University, Uppsala, Sweden. The generation of Zbed6‐null mice has been described in a previous study [14]. All the animals were bred at the Animal Experiment Center of CAAS. Samples of skeletal muscle were collected from 5‐day‐old, 5‐month‐old and 8‐month‐old male wild‐type (WT) and ZBED6‐KO Bama pigs, as well as from 18‐month‐old male mice, for subsequent experiments. The animals were euthanised following the completion of the experiments. Specifically, the Bama pigs were humanely euthanised via a single intravenous injection of sodium pentobarbital at a dose of 200 mg/kg, while the mice were euthanised via the inhalation of excess ether in a sealed chamber. For measuring the muscle weights, the lean tissue was dissected from the left side of the carcass of Bama pigs at different ages and subsequently weighed. The weight of the lean tissue was used as a measure of the weight of the muscle. The hair, head, hooves, tail and internal organs were removed following slaughter. The carcass was then bisected along the dorsal midline into the left and right halves, and the weight of the left half was recorded. For measuring the muscle weights, the lean tissue was dissected from the left side of the carcass of Bama pigs at different ages and subsequently weighed. The weight of the lean tissue represented the weight of the muscle. The muscle ratio was defined as the ratio of the weight of the lean tissue to the total weight of the left side of the carcass. The tissues were rapidly excised, immediately transferred to liquid nitrogen and finally stored at −80°C for subsequent RNA and protein analyses. For histological examination, a portion of the skeletal muscle samples was embedded in Tissue Tek OCT compound, rapidly frozen in isopentane chilled with liquid nitrogen and sectioned into 5–10 μm‐thick slices using a cryostat microtome for subsequent immunofluorescence staining. The remaining muscle tissues were fixed in formalin, embedded in paraffin and stained with haematoxylin and eosin (H&E). Unless otherwise specified, all the pigs and mice used in this study were male, and each experimental group consisted of at least three animals.

### 2.2 Cell Culture and Treatment

The C2C12 cell line (ATCC) was cultured in Dulbecco's modified Eagle medium (DMEM) supplemented with 4.5 g/L glucose (11955065, Gibco, United States), 10% foetal bovine serum (FBS) (10099141C, Gibco, United States) and 1% penicillin–streptomycin solution (P1400, Solarbio, Beijing, China). After the cells reached a confluence of 80%–90%, the differentiation medium was switched to DMEM supplemented with 2% horse serum (16050122, Gibco, United States) and 1% penicillin–streptomycin solution, and the cells were cultured for 72 h. For specific experiments, the cells were treated with an siRNA against Dkk3 (5′‐GGGAAATAACACAGTCCAT‐3′) or adenoviral vectors encoding the ZBED6, Dkk3 and GFP proteins for 12 h, following which they were allowed to fully differentiate over a period of 72 h. The cells were then harvested for analysing gene expression by quantitative polymerase chain reaction (qPCR) and immunoblotting analyses using TRIzol reagent (R711, Vazyme, China) and RIPA (P0013B, Beyotime Biotechnology, China) buffer, respectively. To induce atrophy, the myotubes that had undergone differentiation for 72 h were incubated with 100 μM Dex (HY‐14648, MCE, United States) for 24 h.

The coding sequence of Zbed6 in C2C12 cells was targeted using CRISPR/Cas9 technology. The sequence of the specific guide RNA (sgRNA) for *Zbed6* was 5′‐CACCGGAAGCTAACGAAGCAGGG‐3, which was designed using the CRISPOR program (http://crispor.tefor.net). The sequences of the guide RNA (gRNA) and Cas9 protein were cotransfected into C2C12 cells. The individual cell clones were screened using the limited dilution method, followed by Sanger sequencing of each clone.

Murine primary skeletal muscle satellite cells were isolated from the gastrocnemius muscle (GM) of the hindlimbs of 3‐week‐old mice and cultured as previously described [17]. The muscle tissues were dissected to remove the fascia, minced into a fine paste and mixed with three times the volume of 2% Type I collagenase. The mixture was then incubated at 37°C for 1–2 h with gentle agitation to allow digestion. The growth medium consisted of Ham's F‐10 nutrient mixture (Gibco, United States) supplemented with 20% FBS (10099141C, Gibco, United States) and 2.5 ng/mL bFGF (HY‐P5321, MCE, United States). The differentiation medium consisted of DMEM (Gibco, United States) supplemented with 2% horse serum (16050122, Gibco, United States), and the cells were allowed to differentiate for 72 h. All the media used in the experiments were supplemented with 1% penicillin/streptomycin solution (P1400, Solarbio, Beijing, China).

### 2.3 Others

All details of our methods are shown in Data S1 [15, 18].

## 3 Results

### 3.1 Dkk3 Is a Target of ZBED6 and Potentially Regulates Age‐Related Skeletal Muscle Atrophy

**FIGURE 1:**
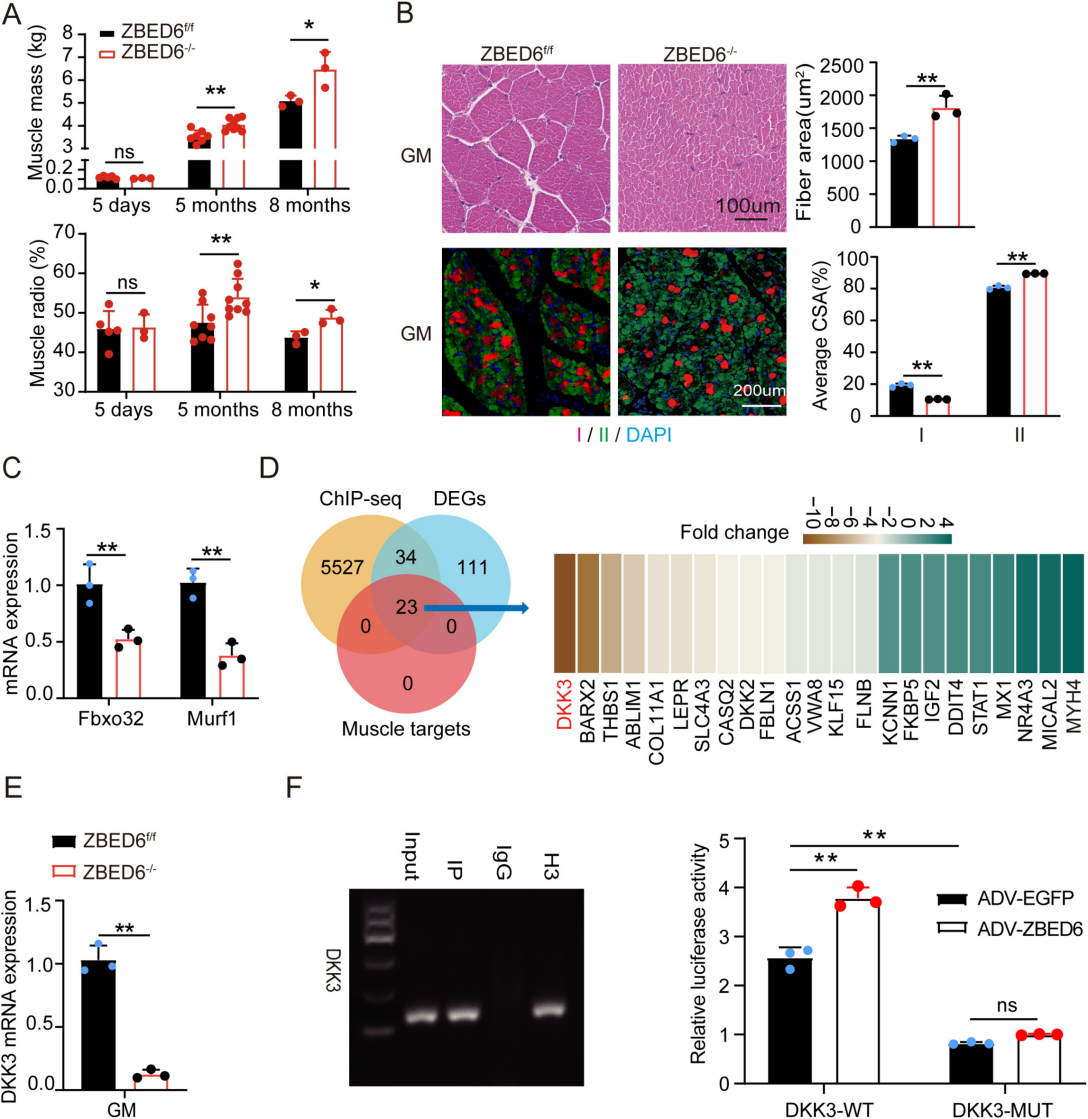
Dkk3 is a potential target gene for ZBED6 to regulate age‐related skeletal muscle atrophy. (A) Change in muscle mass and muscle radio of wild‐type and ZBED6‐KO Bama male pigs (lean mass was dissected from the left carcass and weighed. The lean meat rate was determined by the ratio of lean meat weight and carcass weight of the left carcass). The number of pigs in different periods was five wild‐type pigs and three ZBED6‐KO pigs at 5 days of age; 5 months old wild‐type *n* = 8, ZBED6‐KO pigs *n* = 9; 18 months old wild‐type *n* = 3, ZBED6‐KO pigs *n* = 3. (B) Representative H&E staining, fibre‐type staining and quantification of histological cross sections in GM tissues (scale bar = 100 μm) of ZBED6‐KO and controls in 8‐month‐old Bama pig. Myosin heavy chain type I and IIa (green), IIb (red) and DAPI (blue) (scale bar = 200 μm). Representative images and average myofibre area are shown. *n* = 3. (C) Fbxo32 and Murf1 expression levels of 8 months old pigs. *n* = 3. (D) Integrated analysis of RNA‐seq data of WT and ZBED6‐KO pigs and ZBED6 ChIP‐seq data from skeletal muscle reveals 23 muscle targets; the fold change of target genes was showed in right. (E) Dkk3 mRNA expression level of wild‐type and ZBED6‐KO Bama pigs (8 months old). *n* = 3. (F) ChIP‐PCR agarose gel electrophoresis of Zbed6 binding site in porcine Dkk3 promoter region (Marker I:600, 500, 400 300, 200, 100 bp) and Luciferase analysis showing the effects of overexpressing of ZBED6 on porcine wild‐type Dkk3‐WT luciferase (WT‐ZBS) or mutant Dkk3‐MUT luciferase (MUT‐ZBS). *n* = 3. GAPDH served as internal control. Data are expressed as mean ± SEM; *p < 0.05, **p < 0.01.

The findings revealed that *ZBED6*‐KO significantly increased the muscle mass (17.39%, 27.28% *p* < 0.05) and muscle ratio (13.64%, 11.67% *p* < 0.05) in the adult 5‐ and 8‐month‐old ZBED6‐KO Bama pig model previously generated in our study [15]; however, ZBED6‐KO did not exert any effects on the muscle mass or muscle ratio during the early postnatal stage (5 days after birth) (Figure 1A) and did not exert any effects on the weight of the carcass and the weight of the left carcass at all stages (Figure S2A). These findings demonstrated that ZBED6 exerts age‐related effects on the development of skeletal muscle, which aligns with the findings of previous reports indicating that the transcriptional regulation of ZBED6 is disrupted by an increase in DNA methylation at its binding sites during foetal development [12]. Age‐related muscle atrophy is associated with a reduction in the CSA of myofibres or the selective loss of Type II myofibres [19, 20, 21]. Our findings revealed that the CSA of myofibres and the proportion of Type II myofibres in the GM increased significantly by approximately 35% and 10% (all *p* < 0.01), respectively, in ZBED6‐KO pigs compared to those of WT pigs (Figure 1B, Figure S2B). Consistent with the change in muscle phenotype, the mRNA levels of Fbxo32 and MURF1, which are key biomarkers of muscle atrophy, decreased significantly following the depletion of ZBED6 (Figure 1C).

To explore the underlying regulatory mechanism, the GM tissues from 8‐month‐old ZBED6‐KO and WT pigs were subjected to ChIP‐seq and RNA‐seq analyses, which led to the identification of 5584 target genes and 168 DEGs (Figure 1D, Figure S2C–F, Table S1). A total of 23 target genes of ZBED6 associated with muscle development were identified by overlapping the ChIP‐seq and RNA‐seq data. Among these target genes, the change in gene expression was highest for Dkk3 (fold change = −3.38, *p* < 0.01) (Figure 1E), which plays crucial roles in muscle atrophy and homeostasis [8, 22, 23]. *Dkk3* is a secreted glycoprotein that is encoded by the Dkk gene family (*Dkk*1–4) [24]. Unlike other members of the Dkk family, *Dkk3* is unable to bind to the Lrp and Kremen proteins and is the only member that is enriched in skeletal muscles [25, 26]. ChIP‐PCR analysis using primers designed for the upstream ChIP‐seq peak of *Dkk3* confirmed that *ZBED6* directly binds to *Dkk3* (Figure 1F). Consensus sequence analysis revealed that the conserved ZBED6‐binding site (ZBS) sequence on the *Dkk3* gene was ‘GCTCG’. The ChIP‐seq peak containing the ZBS, which spanned from −5780 to −6138, was cloned into the luciferase vector to generate two recombinant plasmids driven by the WT (WT‐ZBS) or mutant (MUT‐ZBS) peak sequences (Figure S3B). The findings revealed that MUT‐ZBS acted as a repressive element and reduced the luciferase activity to approximately 40%, whereas WT‐ZBS functioned as an activating element, and the luciferase activity was greater than that observed following ZBED6 overexpression (Figure 1F). These results demonstrated that ZBED6 targets Dkk3 to potentially regulate age‐related muscle atrophy in pigs.

### 3.2 Depletion of Zbed6 Attenuates Age‐Induced Muscle Atrophy in Mice

**FIGURE 2:**
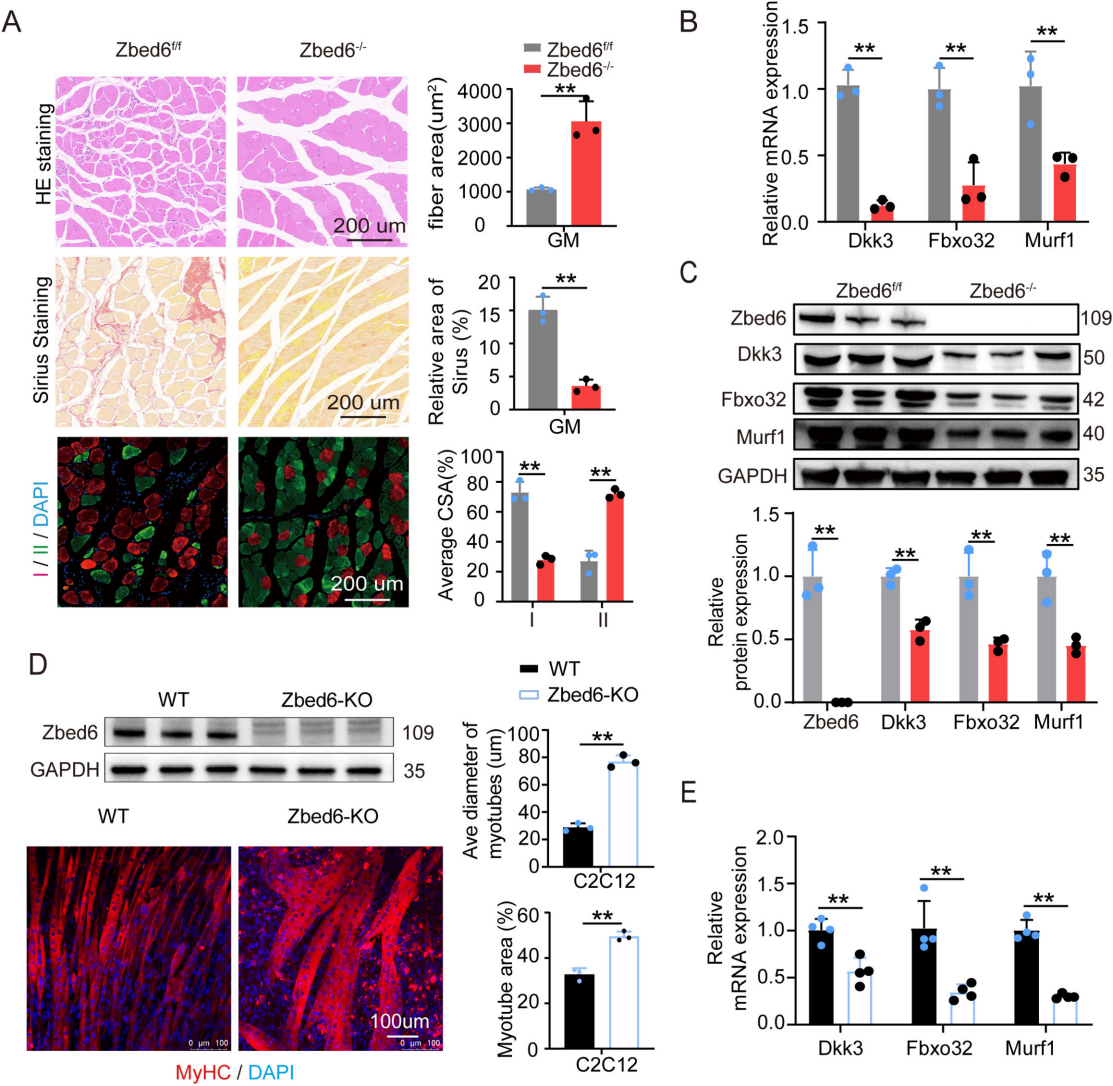
Zbed6 controls Dkk3 transcription in ageing‐induced muscle atrophy. (A) Representative H&E staining (top), Sirius (middle), fibre‐type staining (bottom) and quantification (right) of histological cross sections in GM tissues of Zbed6^−/−^ and controls of 18 months old mice. Myosin heavy chain type I and IIa (red), IIb (green) and DAPI (blue). Representative images are shown. Scale bar = 200 μm. *n* = 3; (B) Dkk3, Fbxo32 and Murf1 mRNA expression levels in mice described in A. *n* = 3. (C) Representative western blotting and quantification of Dkk3, Fbxo32 and Murf1 in mice described in A. GAPDH served as internal control. *n* = 3. (D) Representative western blotting of Zbed6 in WT and Zbed6‐KO C2C12 cells (*n* = 3) and representative images for fibre diameter and area of myotubes (myogenic differentiation takes 72 h). Red indicated myosin heavy chain (MyHC) immunofluorescent staining and DAPI (blue). Scale bars = 100 μm. (E) Dkk3, Fbxo32 and Murf1 mRNA expression levels decreased after Zbed6 depleted. *n* = 4. GAPDH served as internal control. Data are expressed as mean ± SEM; *p < 0.05, **p < 0.01.

Murine models of muscle atrophy are more commonly used than porcine models in studies on age‐related muscle atrophy. This is primarily attributed to the fact that pigs do not exhibit significant signs of ageing until they are over 20 years old, whereas mice can serve as a highly suitable model for studying age‐related skeletal muscle atrophy at the age of 18 months [27, 28]. Previous studies generated Zbed6^−/−^ mouse model and reported that the loss of Zbed6 increased skeletal muscle weight in mice [14]. The present study investigated the phenotypes associated with muscle atrophy in ageing, 18‐month‐old *Zbed6* ^flox/flox^ (*Zbed6* ^fl/fl^) and *Zbed6*‐KO (*Zbed6* ^−/−)^ mice. The results of H&E staining revealed that the myofibres of *Zbed6* ^−/−^ mice were more densely arranged and exhibited a higher average CSA compared with those of *Zbed6* ^fl/fl^ mice (Figure 2A, Figure S3A). Compared to WT mice, *Zbed6*‐KO mice exhibited a 76% reduction in the area of skeletal muscle fibrosis (3.581 ± 1.035% vs. 15.093 ± 2.199% in controls), a hallmark of muscle atrophy [29] (Figure 2A, Figure S3A). Additionally, compared to that of the control group, the proportion of Type II fibres was 50% higher in ageing Zbed6^−/−^ mice (Figure 2A, Figure S3A). The mRNA and protein levels of Dkk3, Fbxo32 and Murf1 were significantly lower in *Zbed*6^−/−^ mice compared to those of *Zbed6* ^fl/fl^ mice (Figure 2B,C). We additionally generated C2C12 cells with *Zbed6*‐KO for assessing the effects of *Zbed6* on myotube atrophy. Consistent with the findings in Zbed6^−/−^ mice, the loss of Zbed6 significantly increased the fusion index and diameter of the myotubes while downregulating the mRNA expression of Dkk3, Fbxo32 and Murf1 in C2C12 cells (Figure 2D,E). Murine primary skeletal muscle satellite cells were additionally isolated in this study. The findings revealed that *Zbed6*‐KO increased the area of the myotubes by approximately 30% compared to that of the normal control (*p* < 0.01). These results suggested that the knockout of *Zbed6* promoted the differentiation of myoblasts in mice (Figure S3C). Additionally, the loss of Zbed6 prevented the ageing of C2C12 cells induced by D‐galactose (Figure S3D). Further analysis of the promoter region of the murine *Dkk3* revealed that Zbed6 could target the ZBS in the first exon of the *Dkk3* gene (Figure S3E). Mutation of the murine ZBS sequence reduced the luciferase activity to approximately 35%, whereas the depletion of *Zbed6* significantly reduced the luciferase activity of the WT sequence (Figure S3E). These results suggested that the loss of *Zbed6* protected against age‐related muscular atrophy in vivo and in vitro, and that *Zbed6* regulates the expression of *Dkk3* by directly binding to its promoter.

### 3.3 Dkk3 Is a Crucial Component in the Protection Conferred by *Zbed6*‐KO Against Muscle Atrophy

**FIGURE 3:**
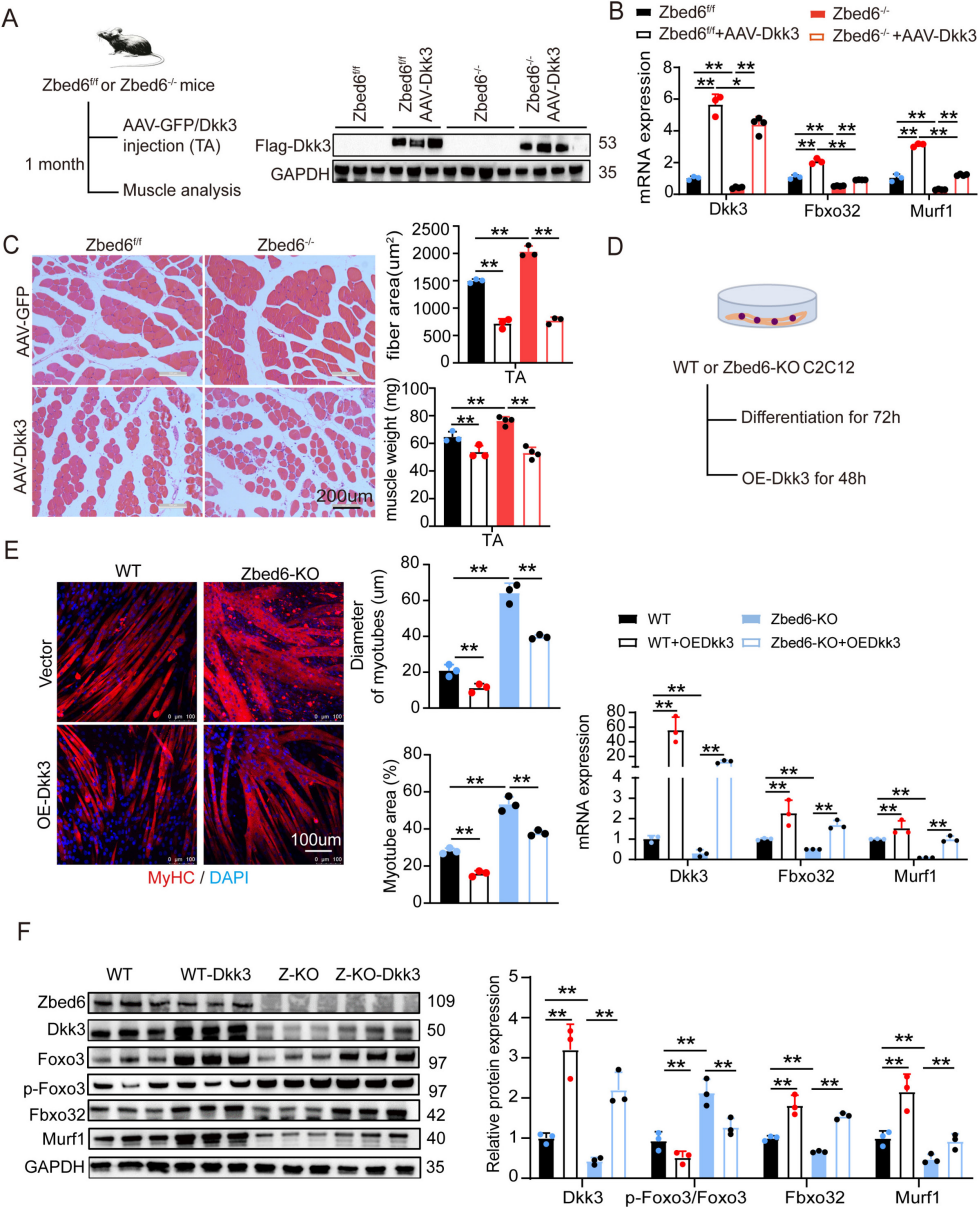
Overexpression of Dkk3 downregulation Zbed6 deficiency‐mediated against muscle atrophy in vivo and vitro. (A) Schematic representation of mouse model. AAV9‐myo2A‐GFP and AAV‐myo2A‐Dkk3 were injected in TA muscle of the left and right legs of the same mice (3‐month‐old) in Zbed6^f/f^ and Zbed6^−/−^ mice for one month, and the following experimental setup (*n* = 3 mice of Zbed6^f/f^; *n* = 4 mice of Zbed6^−/−^) (left). Representative western blotting and quantification of Flag‐Dkk3 (right). (B) Dkk3, Fbxo32 and Murf1 mRNA expression levels in mice described in A. (C) Representative H&E staining (left), average CSA (upper right) and TA muscle weight in mice described in A. Scale bars = 200 μm. (D) Schematic representation of cultured myotubes using 2% horse serum; the differentiation was induced to form myotubes for 72 h and then infected by adenovirus‐encoding Dkk3 or control vector for 48 h. (E) Representative images and quantification (left) for fibre diameter and area of myotubes infected for 120 hours in C2C12 described in D. Red indicated myosin heavy chain (MyHC) immunofluorescent staining and DAPI (blue). Scale bars = 100 μm. Dkk3, Murf1 and Fbxo32 mRNA expression level (right). *n* = 3. (F) Representative western blotting and quantification of Zbed6, Dkk3, Fbxo32, Murf1, phosphorylated protein levels of FoxO3 and total FoxO3 in C2C12 described in D. GAPDH served as internal control. Data are expressed as mean ± SEM; *p < 0.05, **p < 0.01.

We subsequently investigated whether an increase in the expression of *Dkk3* could promote muscle atrophy in Zbed6^−/−^ mice and Zbed6‐KO C2C12 cells. To this end, recombinant AAV‐myo2A‐Dkk3 and AAV‐myo2A‐EGFP vectors were injected into the TA muscles of the left and right legs of 3‐month‐old mice, and samples of TA muscle were collected after 1 month (Figure 3A). Flag‐tagged Dkk3 was subsequently detected by immunoblotting and qRT‐PCR (Figure 3A,B). The results of qRT‐PCR analysis revealed that the overexpression of *Dkk3* restored the mRNA expression of Fbxo32 and Murf1 (Figure 3B). Additionally, the overexpression of *Dkk3* in mice abolished the resistance to muscle atrophy conferred by *Zbed6*‐KO, leading to the formation of smaller myofibres and a reduction in the weight of the TA muscle (Figure 3C). These findings indicated that the knockout of *Zbed6* protected against muscle atrophy by downregulating the expression of *Dkk3* in mice. The results of MyHC immunostaining revealed that the knockout of *Zbed6* significantly increased the fusion index and diameter of the myotubes compared to those of the WT control C2C12 cells. However, the overexpression of *Dkk3* counteracted the protective effect conferred by *Zbed6*‐KO on myotube atrophy and resulted in the formation of thinner myotubes (Figure 3D,E). This suggested that the downregulation of *Dkk3* is crucial for the protection conferred by *Zbed6*‐KO against muscle atrophy. Mechanistically, the loss of *Zbed6* suppressed the activation of Dkk3, which in turn increased the inhibitory phosphorylation of *FoxO3* and decreased the total protein levels. The downregulation of *FoxO3* expression and the increased phosphorylation of FoxO3 further downregulated the expression of *Murf1* and *Fbxo32*, and the overexpression of Dkk3 counteracted the effects of *Zbed6*‐KO on FoxO3 (Figure 3F). These findings demonstrated that the loss of *Zbed6* attenuated muscular atrophy by downregulating the Dkk3‐FoxO3 axis both in vivo and in vitro.

### 3.4 Loss of *Zbed6* Confers Protection Against Dex‐Induced Muscle Atrophy

**FIGURE 4:**
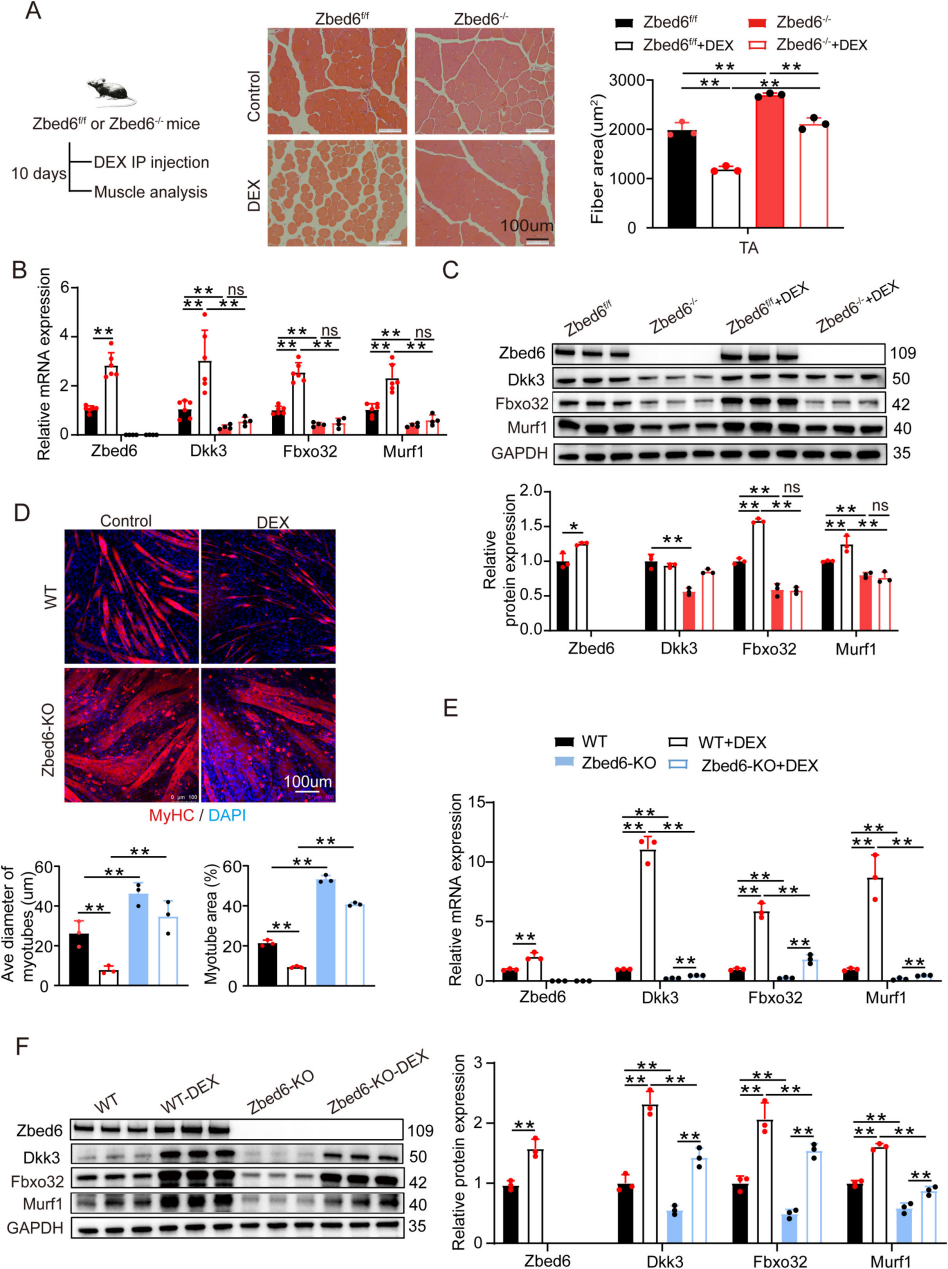
Zbed6 depletion protects dexamethasone‐induced myotubes and muscles atrophy. (A) Schematic representation of Dex injection, representative H&E staining and quantification of histological cross sections derived from Zbed6^−/−^ and controls (3‐month‐old) TA muscles, and mice was injected with or without 15 mg/kg/day of Dex for 10 days. Scale bars = 100 μm. *n* = 6. (B) mRNA expression levels of Zbed6, Dkk3, Murf1 and Fbxo32 described in A. *n* = 6. (C) Representative western blotting and quantification of Zbed6, Dkk3, Murf1 and Fbxo32 in mice described in A. *n* = 3. (D) Representative images (top) and quantification (bottom) for fibre diameter and area of myotubes. WT and Zbed6‐KO C2C12 myotubes with or without Dex; the myotubes were treated with 100 μM Dex for 24 h. Red indicated myosin heavy chain (MyHC) immunofluorescent staining and DAPI (blue). Scale bars = 100 μm. *n* = 3. (E, F) mRNA and protein expression levels of Zbed6, Dkk3, Murf1 and Fbxo32 described in D. *n* = 3. GAPDH served as internal control. Data are expressed as mean ± SEM; *p < 0.05, **p < 0.01.

The role of Zbed6 in glucocorticoid‐induced muscle atrophy was further investigated in this study. Elevated glucocorticoid levels are a major factor in skeletal muscle atrophy [9, 10, 11]. However, the role of Dkk3 in regulating glucocorticoid‐induced skeletal muscle atrophy remains unexplored. In this study, C2C12 myotubes were treated with the glucocorticoid Dex, which significantly increased the mRNA and protein levels of Zbed6, Dkk3, Fbxo32 and Murf1 (Figure S4A,B). To determine whether *Zbed6*‐KO protects against Dex‐induced skeletal muscle atrophy, Dex was administered to Zbed6^fl/fl^ and Zbed6^−/−^ mice for 10 days, and the resulting muscle phenotypes were examined. Treatment with Dex decreased the CSA of the myofibers in Zbed6^fl/fl^ mice by 40% (*p* < 0.01). However, *Zbed6*‐KO mitigated this reduction and restored the CSA to 106% in Zbed6^−/−^ mice post‐treatment compared to that of untreated WT mice (Figure 4A, Figure S4C). Consistent with the phenotypic and histological findings, Zbed6^−/−^ mice were resistant to the induction of Dkk3, Fbxo32 and Murf1 following treatment with Dex (Figure 4B,C). As in vivo studies suggested that the depletion of *Zbed6* protected myofibres against Dex‐induced atrophy, we determined whether Zbed6‐KO C2C12 cells were resistant to Dex‐induced reduction in myotube size. The diameter of the myotubes in WT cells decreased significantly after 24 h of treatment with Dex; however, there were no significant changes in the Zbed6‐KO cells (Figure 4D). Additionally, the loss of *Zbed6* weakened the Dex‐induced upregulation of Dkk3, Fbxo32 and Murf1 (Figure 4E,F). These findings demonstrated that the depletion of *Zbed6* attenuated Dex‐induced muscle atrophy both in vitro and in vivo and emphasised the critical role of Dkk3 in this process.

### 3.5 Dkk3 Is a Critical Component in the Protective Effect Conferred by *Zbed6*‐KO Against Dex‐Induced Muscle Atrophy

**FIGURE 5:**
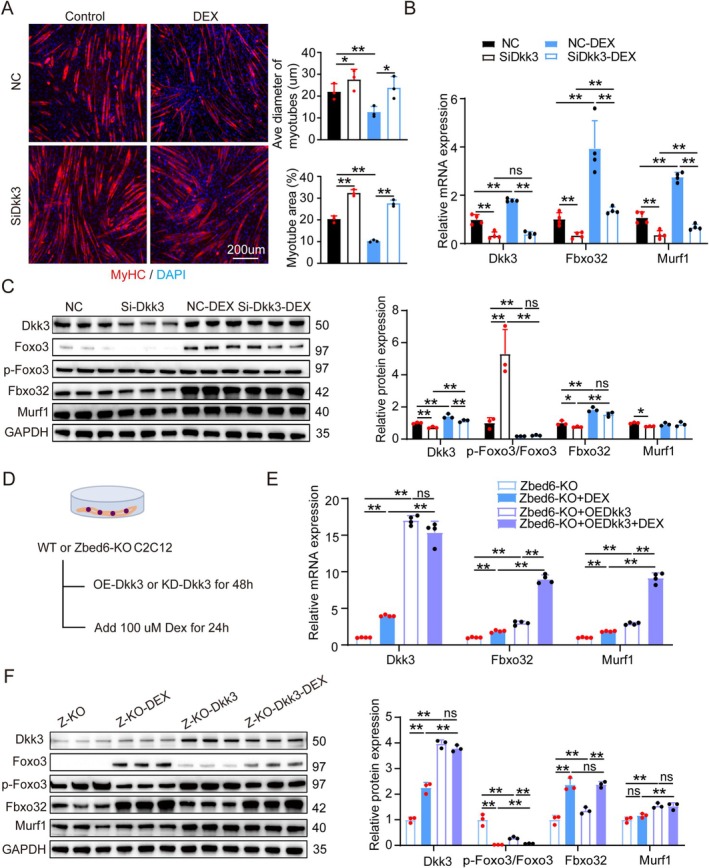
Dkk3 mediates Dex‐induced muscle atrophy regulated by Zbed6. (A) Representative images (left) and quantification (right) for fibre diameter and area of myotubes knockdown Dkk3; myotubes were treated with Dex. Primary myotubes were infected by Si‐Dkk3, 48 h after infection, and the myotubes were treated with 100 μM Dex for 24 h. Red indicated myosin heavy chain (MyhC) immunofluorescent staining and DAPI (blue). Scale bars = 200 μm. (B) Dkk3, Fbxo32 and Murf1 mRNA expression levels in myotubes described in A. *n* = 4. (C) Representative western blotting and quantification of Dkk3, Fbxo32, Murf1, phosphorylated protein levels of Foxo3 and total FoxO3 in myotubes described in A. *n* = 3. (D) Schematic representation of WT and Zbed6‐KO myotubes with overexpression Dkk3 or knockdown Dkk3, 48 h after infection, the myotubes treated with or without 100 μM Dex for 24 h. (E) Dkk3, Fbxo32 and Murf1 mRNA expression level of Zbed6‐KO myotubes with overexpression Dkk3. *n* = 4. (F) Representative western blotting and quantification of Dkk3, Fbxo32, Murf1, phosphorylated protein levels of FoxO3 and total FoxO3 of Zbed6‐KO myotubes with overexpression Dkk3. *n* = 3. GAPDH served as internal control. Data are expressed as mean ± SEM; *p < 0.05, **p < 0.01.

To further investigate the role of Dkk3 in Dex‐induced muscle atrophy, we performed *Dkk3* knockdown in cultured myotubes. After 48 h of knockdown, the myotubes were treated with Dex for 24 h. The findings revealed that *Dkk3* knockdown protected myotubes from Dex‐induced atrophy, and the expression levels of Zbed6, Dkk3, Fbxo32, Murf1 and FoxO3/p‐FoxO3 were downregulated compared to those of the controls (Figure 5A–C). To investigate the role of Dkk3 in the resistance to Dex‐induced muscle atrophy conferred by Zbed6‐KO, *Dkk3* was overexpressed in WT and Zbed6‐KO myotubes, and atrophy was subsequently induced using Dex (Figure 5D). The overexpression of *Dkk3* in both the WT and Zbed6‐KO myotubes exacerbated Dex‐induced muscle atrophy; upregulated the levels of FoxO3, Fbxo32 and Murf1; and reduced the phosphorylation of FoxO3 (Figure 5E–F, Figure S5A–C). These findings indicated that the depletion of *Zbed6* protected the myotubes from Dex‐induced atrophy by downregulating the expression of Dkk3. Altogether, these findings demonstrated that Dkk3 plays a critical role in the protective effect conferred by Zbed6‐KO against Dex‐induced muscle atrophy.

### 3.6 *Zbed6* Overexpression Promoted Muscle Atrophy in Myotubes and Young Mice

**FIGURE 6:**
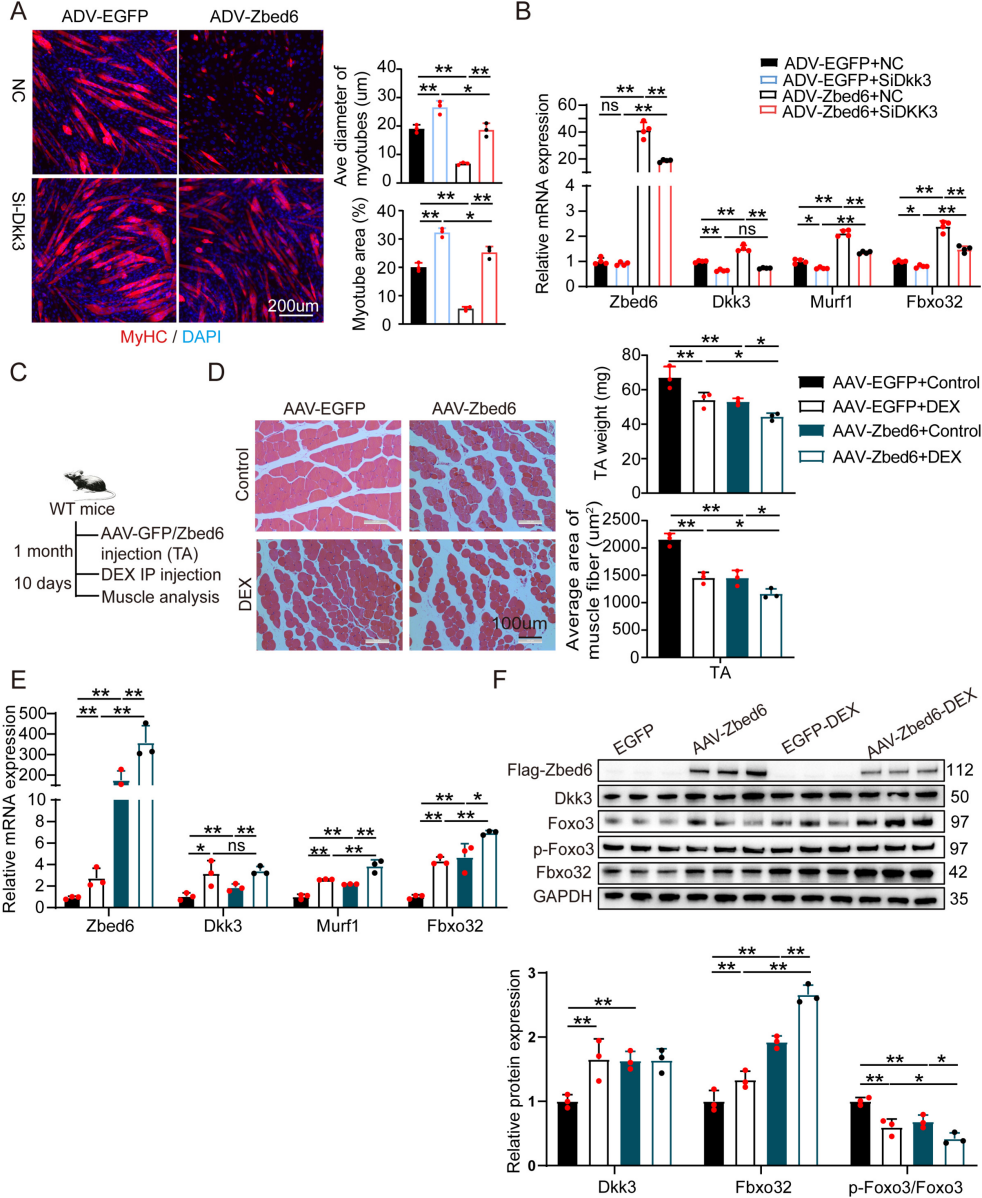
Overexpression of Zbed6 accelerates muscle atrophy in vitro and vivo*.* (A) Representative images (left) and quantification (right) of myotubes. Primary myotubes overexpressing Zbed6 by adenovirus‐encoding Zbed6 or control vector, 24 h after infection, the myotubes infection with Si‐Dkk3 or negative‐control (NC). Red indicated myosin heavy chain (MyhC) immunofluorescent staining and DAPI (blue). Scale bars = 200 μm. *n* = 3. (B) Zbed6, Dkk3, Murf1 and Fbxo32 mRNA expression level in C2C12 described in A. *n* = 4. (C) Schematic representation of virus and Dex injection. AAV9‐myo2A‐GFP and AAV‐myo2A‐Zbed6 were injected in TA muscle of the left and right legs of the same mice (3‐month‐old). One month later, the mice were intraperitoneally injected with or without 15 mg/kg/day of dexamethasone for 10 days. *n* = 3. (D) Representative H&E staining (left) and quantification (right) of histological cross sections derived from TA muscles and TA muscle weight described in C. Scale bars = 100 μm. (E) Zbed6, Dkk3, Fbxo32 and Murf1 mRNA expression levels described in C. *n* = 3. (F) Representative western blotting and quantification of Dkk3, Fbxo32, Murf1, phosphorylated protein levels of FoxO3 and total FoxO3 in muscle described in C. *n* = 3. GAPDH served as internal control. Data are expressed as mean ± SEM; *p < 0.05, **p < 0.01.

To investigate whether elevated levels of Zbed6 can induce muscle atrophy, the myotubes were infected with adenovirus encoding Zbed6 and EGFP, 24 h prior to transfection with SiDkk3 or the control. The findings revealed that the overexpression of *Zbed6* exacerbated muscle atrophy, decreased the diameter of the myotubes (Figure 6A) and increased the expression levels of Dkk3, Fbxo32 and Murf1 (Figure 6B). Treatment with Si‐Dkk3 reversed the exacerbated muscle atrophy and downregulated the expression of Fbxo32 and Murf1 in myotubes overexpressing Zbed6 (Figure 6A,B).

Zbed6 was specifically overexpressed in the TA muscles of mice using an engineered AAV vector, followed by treatment with Dex. Specifically, recombinant AAV‐myo2A‐Zbed6 and AAV‐myo2A‐EGFP vectors were injected into the contralateral TA muscles of 3‐month‐old mice for 1 month, following which the mice were treated with Dex for 10 days (Figure 6C). The overexpression of *Zbed6* significantly reduced the weight of the TA muscles and CSA of the myofibres, and treatment with Dex further exacerbated muscle atrophy (Figure 6D). Consistent with these phenotypic changes, the overexpression of *Zbed6* and treatment with Dex significantly upregulated the mRNA and protein expression of Zbed6, Fbxo32 and Murf1 (Figure 6E,F). These observations suggest that the ectopic expression of Zbed6 in myotubes and mice is sufficient to induce muscle atrophy, which further indicates that Dkk3 is a critical mediator of Zbed6‐induced muscle atrophy.

## 4 Discussion

Skeletal muscle atrophy is significantly influenced by gene regulation in response to ageing and elevated serum glucocorticoid levels; however, the precise underlying mechanisms remain unclear [30]. By employing porcine and murine models of muscle atrophy, the present study was aimed at elucidating the critical role of ZBED6 in preserving myofibre size during age‐ and glucocorticoid‐induced muscle atrophy. Notably, our findings demonstrated that the depletion of *ZBED6* attenuated muscle atrophy by downregulating the expression of *Dkk3*, a key regulator of muscular atrophy, by directly binding to its promoter region. These findings suggested that *ZBED6* can potentially modulate age‐ and Dex‐induced muscle atrophy by targeting *Dkk3*.

Previous studies have demonstrated that *ZBED6* significantly modulates the growth of skeletal muscle in placental mammals by targeting IGF2 [13, 14]. Additionally, *ZBED6*‐KO is associated with elevated muscle growth rates and higher muscle mass in pigs and mice [14, 15] and has been shown to protect against sepsis‐induced muscular atrophy in pigs by targeting DOCK3 [16]. However, the precise mechanisms underlying the effects of ZBED6 on age‐ and Dex‐induced muscle atrophy are yet to be fully understood. Our results demonstrated that the loss of *ZBED6* promoted muscle growth and conferred protection against muscle atrophy induced by ageing and Dex through the downregulation of *Dkk3* expression.

Dkk3 plays a critical role in age‐related muscle atrophy and the differentiation of myoblasts. The knockdown of *Dkk3* in sarcopenic muscles has been shown to restore myofibre size and enhance muscular contractions [8, 22]. During ageing, Dkk3 activates the transcription of Fbxo32 and Murf1 by recruiting FoxO3, and this process depends on elevated concentrations of Dkk3 [8]. We hypothesised that the depletion of Zbed6 suppresses the recruitment of FoxO3 to the promoter regions of *Fbxo32* and *Murf1* by downregulating the expression of *Dkk3* and inhibiting the Dkk3‐FoxO3‐Fbxo32 axis, thereby mitigating muscle loss. On one hand, ZBED6 downregulates the expression of the FoxO3 protein, while on the other hand, it promotes the phosphorylation of FoxO3. This mechanism is critical for maintaining muscle mass during ageing and stressful conditions. Our findings highlight that ZBED6 can serve as a potential therapeutic target for muscle atrophy and provides novel insights for the preservation of muscle tissue.

Analysis of the targets regulated by ZBED6 led to the identification of nine genes related to muscle growth that were significantly upregulated following *ZBED6*‐KO in pigs. Among these DEGs, *MYH4* displayed the highest fold change in expression (log2FC = 2.35, *p* < 0.01). Similar results were observed in mice and C2C12 cells following *Zbed6*‐KO, in that the expression of MYH4 was significantly upregulated by approximately ninefold following *ZBED6*‐KO, but downregulated following the overexpression of *ZBED6* (Figure S6A,B). The *MYH1*, *MYH2* and *MYH4* genes encode Type II myofibres, which constitute the majority (> 70%) of myofibres in adult mammals and represent the predominant fibre type reduced in muscle wasting induced by diseases and ageing [31]. The upregulation of *MYH4* helps maintain the integrity of myotubes against Dex‐induced muscle atrophy [32]. Furthermore, *ZBED6*‐KO has been shown to mitigate the loss of Type II myofibres induced by sepsis in pigs [16]. The present study revealed that the mRNA expression levels of MYH1, MYH2 and MYH4 increased significantly, while that of MYH7 was downregulated in ZEBD6‐deficient pigs and mice (Figure S6). The proportion of Type II myofibres in adult pigs and aged mice with *ZBED6*‐KO was approximately 10% and 45% higher, respectively, than those of WT pigs and mice (Figures 1B and 2A). These findings indicated that MYH4 plays a key role in attenuating the muscular atrophy induced by ageing and Dex in *Zbed6*‐KO mice.

Dex is regularly administered to alleviate cerebral oedema and provide symptomatic relief [33]. However, the chronic use of Dex leads to dose‐dependent immunosuppression and severe side effects, including impaired glucose metabolism, reduced muscle strength and decreased insulin sensitivity [34, 35]. These adverse effects are partly attributed to the loss of muscle mass, as skeletal muscles are responsible for the majority of postprandial glucose uptake [36]. The depletion of *Zbed6* decreases insulin production by inhibiting the survival and proliferation of beta cells [37], and Dkk3 is essential for maintaining glucose homeostasis [22]. The present study revealed that the deletion of Zbed6 inhibited Dex‐induced muscle atrophy by downregulating the expression of *Dkk3*. However, treatment with Dex following *Zbed6*‐KO or *Dkk3* knockdown increased the expression of Dkk3, suggesting that Dex may also regulate the expression of Dkk3 via other pathways, which warrants further investigation. Additionally, the upregulation of *Dkk3* expression significantly inhibited the AKT–mTOR pathway, which plays a critical regulatory role in Dex‐induced skeletal muscle atrophy [11, 22]. Increased AKT–mTOR signalling inactivates FoxO3, thereby protecting against muscle atrophy [38]. Altogether, these findings suggest that the deletion of *ZBED6* can prevent Dex‐induced skeletal muscle atrophy by preserving the insulin pathway. Previous studies have demonstrated that *Zbed6*‐KO also protects against obesity and insulin sensitivity induced by high‐fat diets [15, 37], suggesting that it may also attenuate obesity‐ or T2D‐induced muscle atrophy. The inhibitory effects of *ZBED6*‐KO on obesity and muscle atrophy may also mitigate the muscle atrophy induced by existing drugs used for the treatment of obesity, providing potential insights for the development of novel therapeutic targets for obesity and diabetes. However, further research is necessary for understanding the contributions of *ZBED6* in obesity‐induced muscle atrophy.

Ageing, obesity and T2D can suppress muscle regeneration [39]. The present study revealed that the depletion of Zbed6 promoted the development of myotubes and myofibres, thus conferring protection against age‐induced muscle atrophy. Conversely, the overexpression of *Zbed6* in stable C2C12 cells prevented their differentiation into multinucleate myotubes. A recent study reported that Zbed6 targets gene families that are crucial for skeletal muscle regeneration, including the *Pax* and *Sox* gene families [40], indicating its potential role in muscle regeneration and the maintenance of satellite cells.

Altogether, the present study demonstrated that the depletion of *Zbed6* can mitigate ageing‐ and Dex‐induced muscle atrophy and that *Zbed6*‐KO affects muscle homeostasis via the Dkk3‐FoxO3‐Fbxo32 axis. However, this study is limited by potential interference from other tissues and the need to explore the regulatory effects of Zbed6 on other forms of muscle atrophy and regeneration. The systemic roles of Zbed6 in mice with tissue‐specific *Zbed6*‐KO should be explored in future research.

## Ethics Statement

The manuscript authors certify that they comply with the ethical guidelines for authorship and publishing in the *Journal of Cachexia, Sarcopenia and Muscle*.

## Conflicts of Interest

The authors declare no conflicts of interest.

## Supporting information


**Figure S1**. Schematic of sample collection and experimental design. (A) Phenotypic characterisation of pigs and mice and mechanism analysis. Using pig and mouse models, we determined that ZBED6 knockout inhibits age‐related skeletal muscle atrophy by sacrificing animal models of different ages. ChIP‐seq and RNA‐seq were used to identify Dkk3 as the target gene with the most significant fold‐change. ZBED6 knockout may mitigate age‐related skeletal muscle atrophy by targeting and downregulating Dkk3, thereby mediating the downregulation of the FoxO3‐Fbxo32/MURF1 pathway. (B) Animal experiment design and result explanation. (1) By intraperitoneally injecting dexamethasone into 3‐month‐old Zbed6^f/f^ and Zbed6^−/−^ mice, we found that Zbed6 knockout inhibits dexamethasone‐induced muscle atrophy by downregulating Dkk3. (2) By injecting AAV9‐myo2A‐Dkk3 and AAV9‐myo2A‐GFP into the tibialis anterior muscle of Zbed6^f/f^ and Zbed6^−/−^ mice, we showed that overexpression of Dkk3 induces muscle atrophy in Zbed6^−/−^ mice, further confirming that Dkk3 is a downstream target gene regulated by Zbed6 in muscle atrophy. (3) By overexpressing Zbed6 in the tibialis anterior muscle of wild‐type mice and injecting dexamethasone intraperitoneally after 1 month, we found that overexpression of ZBED6 led to muscle atrophy in the tibialis anterior and exacerbated dexamethasone‐induced muscle atrophy. A series of in vitro experiments demonstrated that the Zbed6‐Dkk3‐FoxO3‐Fbxo32/Murf1 pathway is a key mechanism regulating skeletal muscle atrophy in mice. (C) Cell experiment design and result explanation. (1) After 72 h of myogenic differentiation of WT and Zbed6‐KO cells, dexamethasone was added, and it was found that Zbed6 knockout targeted and downregulated Dkk3, inhibiting dexamethasone‐induced myotube atrophy. (2) After 72 h of myogenic differentiation of WT and Zbed6‐KO cells, overexpression or knockdown of Dkk3 was performed, followed by dexamethasone treatment, which further confirmed that Dkk3 is a downstream target of ZBED6 in regulating myotube atrophy. (3) Knockdown of Dkk3 after ZBED6 overexpression demonstrated that Dkk3 knockdown could alleviate the inhibitory effect of ZBED6 overexpression on myotube differentiation.

**Figure S2**. Porcine phenotype and ChIP‐seq data of ZBED6. (A) The carcass weight and left carcass weight of wild‐type and ZBED6‐KO Bama male pigs. The number of pigs in different periods was five wild‐type pigs and three ZBED6‐KO pigs at 5 days of age; 5 months old wild‐type *n* = 8, ZBED6‐KO pigs *n* = 9; 8 months old wild‐type *n* = 3, ZBED6‐KO pigs *n* = 3. (B) Representative H&E staining and fibre‐type staining of histological cross sections in GM tissues (scale bar = 200 μm) of ZBED6‐KO and controls in 8‐month‐old Bama pig. Myosin heavy chain type I and IIa (red), IIb (green) and DAPI (blue) (scale bar = 400 μm). Representative images and average myofibre area are shown. *n* = 3. (C) Aggregated line plots (top) and heatmaps (bottom) of chromatin immunoprecipitation followed by sequencing (ChIP‐seq) signals over full‐length ZBED6 in Bama pig skeletal muscle. (D) Chromosomal diagram of ZBED6 binding site. (E) ZBED6 binding site ratio. (F) The number of peaks and genes of ZBED6 binding sites.

**Figure S3**. Zbed6 controls Dkk3 transcription in ageing‐induced muscle atrophy. (A) Representative H&E staining (top), Sirius (middle) and fibre‐type staining (bottom) of histological cross sections in GM tissues of Zbed6^−/−^ and controls of 18 months old mice. Myosin heavy chain type I and IIa (red), IIb (green) and DAPI (blue). Representative images are shown. Scale bar = 400 μm. *n* = 3. (B) The wild‐type and ZBS mutant sequences of pigs are indicated. (C) Representative images and quantification for myotubes area of primary skeletal muscle satellite cells from Zbed6^−/−^ and controls. Red indicated myosin heavy chain (MyHC) immunofluorescent staining and DAPI (blue). Scale bars = 200 μm. *n* = 3. (D) Representative images of SA‐β‐gal staining in D‐gal‐induced C2C12 myoblasts of WT and Zbed6‐KO cells. Scale bars, 200 μm. (E) The wild‐type and ZBS mutant sequences of mice are indicated. Luciferase analysis showing the effects of WT and Zbed6‐KO C2C12 cells on mice wild‐type Dkk3‐ZBS luciferase (ZBS‐LUC) or mutant Dkk3‐ZBS luciferase (mZBS‐LUC). Data are expressed as mean ± SEM; *p < 0.05, **p < 0.01.

**Figure S4**. Zbed6 depletion protects dexamethasone‐induced myotubes and muscles atrophy. (A) Zbed6, Dkk3, Fbxo32 and Murf1 mRNA expression level in Dex‐induced atrophy model of C2C12 myotubes (C_(Dex)_ = 0, 10, 20, 50, 100 μM). (B) Representative western blotting and quantification of Zbed6, Dkk3, Fbxo32 and Murf1 in Dex‐induced atrophy model of C2C12 myotubes. (C) The body weight of Zbed6^−/−^ and controls (3‐month‐old) for injected with or without 15 mg/kg/day of Dex for 10 days. GAPDH served as internal control. Data are expressed as mean ± SEM; *p < 0.05, **p < 0.01.

**Figure S5**. Dkk3 mediates Dex‐induced muscle atrophy regulated by Zbed6. (A) Zbed6, Dkk3, Fbxo32 and Murf1 mRNA expression level of WT myotubes with overexpression Dkk3. Representative western blotting (B) and quantification (C) of Dkk3, Fbxo32, Murf1, phosphorylated protein levels of FoxO3 and total FoxO3 of WT myotubes with overexpression Dkk3. *n* = 3. GAPDH served as internal control. Data are expressed as mean ± SEM; *p < 0.05, **p < 0.01.

**Figure S6**. The mRNA expression of Myh4 in pigs and mice. (A) Myh4 mRNA expression level of WT and Zbed6‐KO C2C12 myotubes. *n* = 4. (B) MYH1, MYH2, MYH4 and MYH7 mRNA expression level of wild‐type and ZBED6‐KO Bama pigs (8‐month‐old). *n* = 3. (C) Myh1, Myh2, Myh4 and Myh7 mRNA expression level of Zbed6^f/f^ and Zbed6^−/−^ mice (18‐month‐old). *n* = 3. GAPDH served as internal control. Data are expressed as mean ± SEM; *p < 0.05, **p < 0.01.

**Table S1**. Supporting Information

**Data S1**. Supporting Information
