# Supplementary figures and images for: ZBED6 Knockout Prevents Ageing‐ and Dexamethasone‐Induced Muscle Atrophy via Dkk3 in Pig and Mice
Source: J Cachexia Sarcopenia Muscle. 2025 Jun 4;16(3):e13829. doi: 10.1002/jcsm.13829 (PMC12134784; doi:10.1002/jcsm.13829)

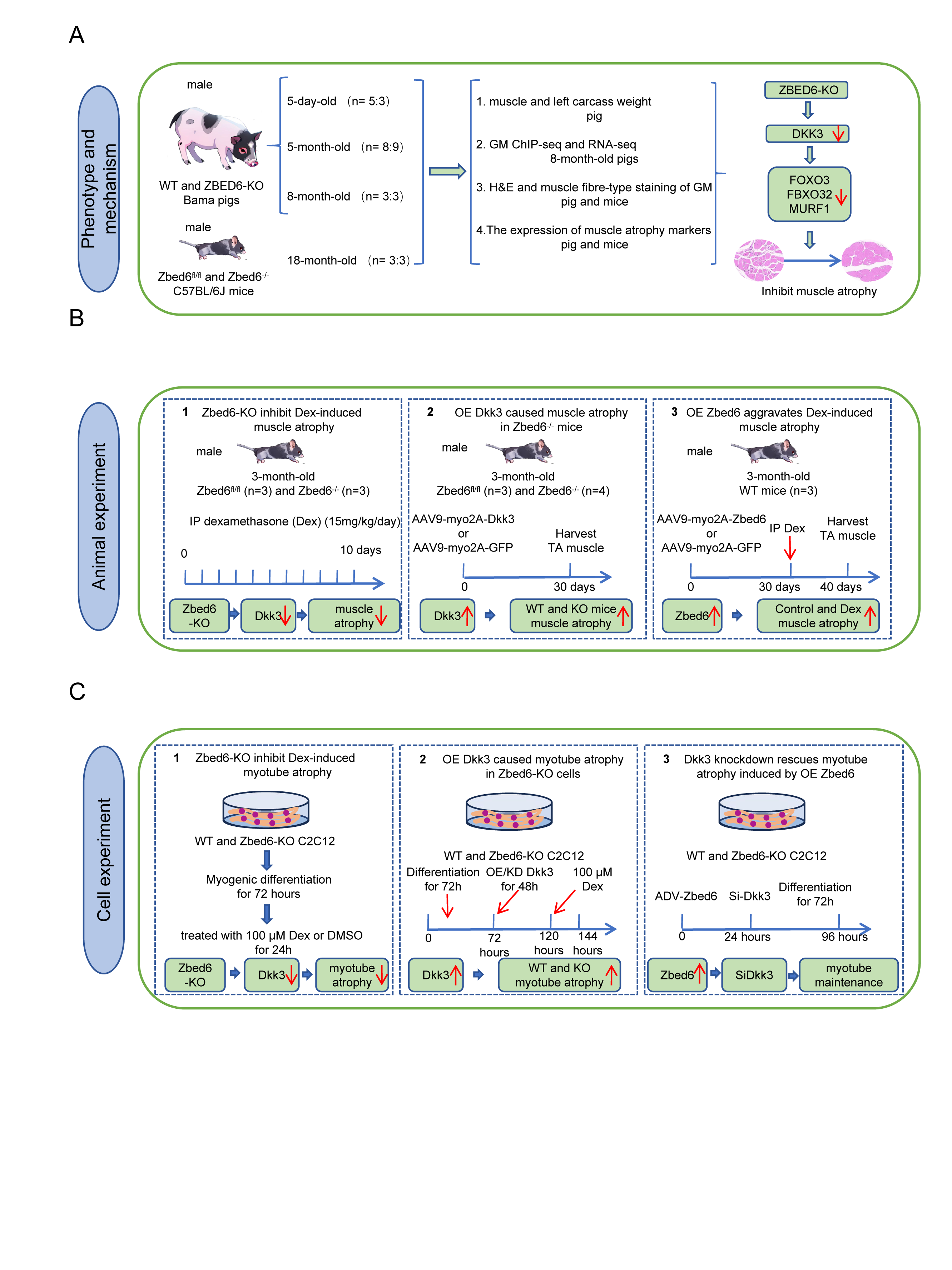

Supplement: Supplementary file 1 — Figure S1. Schematic of sample collection and experimental design. (A) Phenotypic characterisation of pigs and mice and mechanism analysis. Using pig and mouse models, we determined that ZBED6 knockout inhibits age‐related skeletal muscle atrophy by sacrificing animal models of different ages. ChIP‐seq and RNA‐seq were used to identify Dkk3 as the target gene with the most significant fold‐change. ZBED6 knockout may mitigate age‐related skeletal muscle atrophy by targeting and downregulating Dkk3, thereby mediating the downregulation of the FoxO3‐Fbxo32/MURF1 pathway. (B) Animal experiment design and result explanation. (1) By intraperitoneally injecting dexamethasone into 3‐month‐old Zbed6f/f and Zbed6−/− mice, we found that Zbed6 knockout inhibits dexamethasone‐induced muscle atrophy by downregulating Dkk3. (2) By injecting AAV9‐myo2A‐Dkk3 and AAV9‐myo2A‐GFP into the tibialis anterior muscle of Zbed6f/f and Zbed6−/− mice, we showed that overexpression of Dkk3 induces muscle atrophy in Zbed6−/− mice, further confirming that Dkk3 is a downstream target gene regulated by Zbed6 in muscle atrophy. (3) By overexpressing Zbed6 in the tibialis anterior muscle of wild‐type mice and injecting dexamethasone intraperitoneally after 1 month, we found that overexpression of ZBED6 led to muscle atrophy in the tibialis anterior and exacerbated dexamethasone‐induced muscle atrophy. A series of in vitro experiments demonstrated that the Zbed6‐Dkk3‐FoxO3‐Fbxo32/Murf1 pathway is a key mechanism regulating skeletal muscle atrophy in mice. (C) Cell experiment design and result explanation. (1) After 72 h of myogenic differentiation of WT and Zbed6‐KO cells, dexamethasone was added, and it was found that Zbed6 knockout targeted and downregulated Dkk3, inhibiting dexamethasone‐induced myotube atrophy. (2) After 72 h of myogenic differentiation of WT and Zbed6‐KO cells, overexpression or knockdown of Dkk3 was performed, followed by dexamethasone treatment, which further confirmed that Dkk [file JCSM-16-e13829-s005.tif]

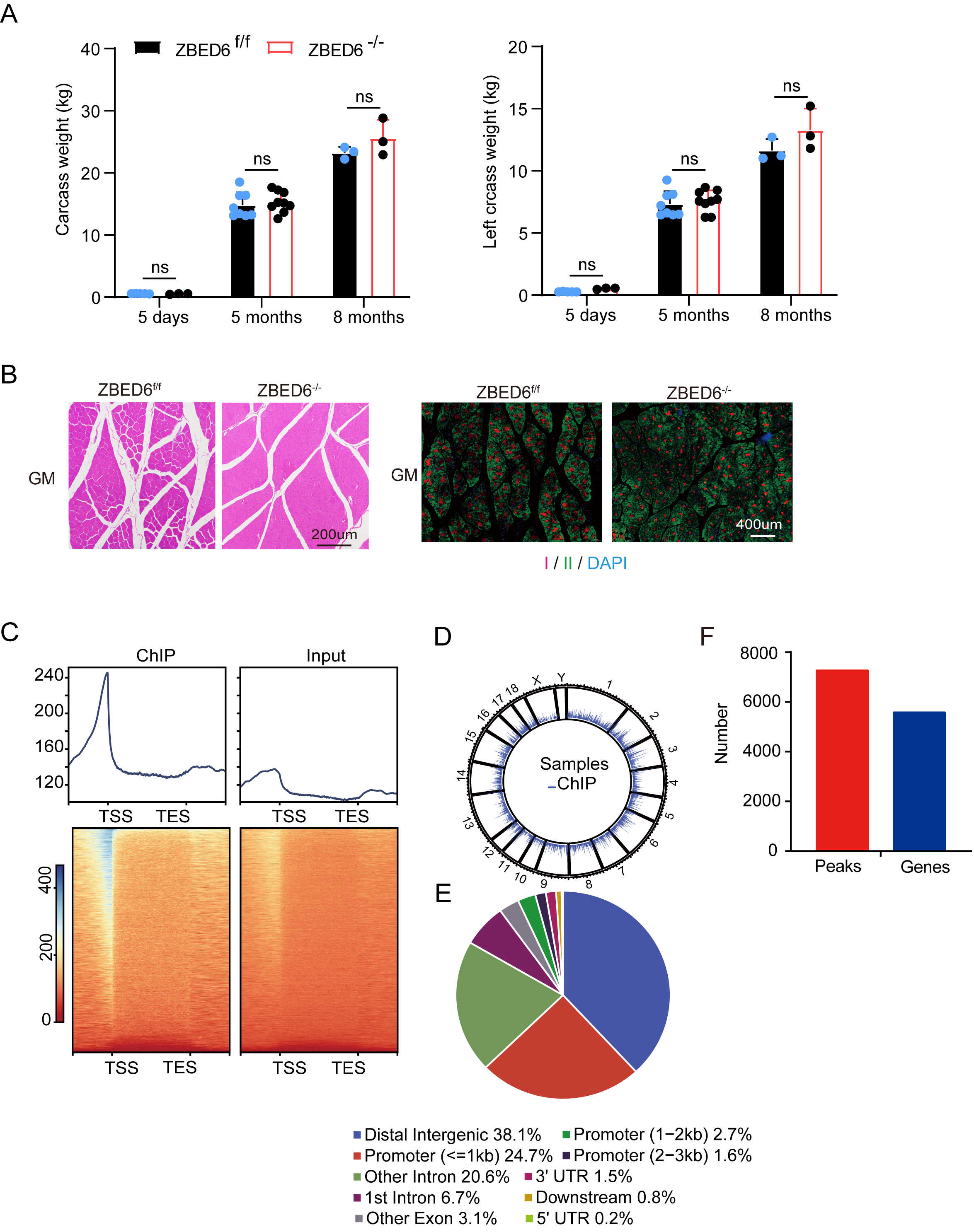

Supplement: Supplementary file 2 — Figure S2. Porcine phenotype and ChIP‐seq data of ZBED6. (A) The carcass weight and left carcass weight of wild‐type and ZBED6‐KO Bama male pigs. The number of pigs in different periods was five wild‐type pigs and three ZBED6‐KO pigs at 5 days of age; 5 months old wild‐type n = 8, ZBED6‐KO pigs n = 9; 8 months old wild‐type n = 3, ZBED6‐KO pigs n = 3. (B) Representative H&E staining and fibre‐type staining of histological cross sections in GM tissues (scale bar = 200 μm) of ZBED6‐KO and controls in 8‐month‐old Bama pig. Myosin heavy chain type I and IIa (red), IIb (green) and DAPI (blue) (scale bar = 400 μm). Representative images and average myofibre area are shown. n = 3. (C) Aggregated line plots (top) and heatmaps (bottom) of chromatin immunoprecipitation followed by sequencing (ChIP‐seq) signals over full‐length ZBED6 in Bama pig skeletal muscle. (D) Chromosomal diagram of ZBED6 binding site. (E) ZBED6 binding site ratio. (F) The number of peaks and genes of ZBED6 binding sites. [file JCSM-16-e13829-s003.tif]

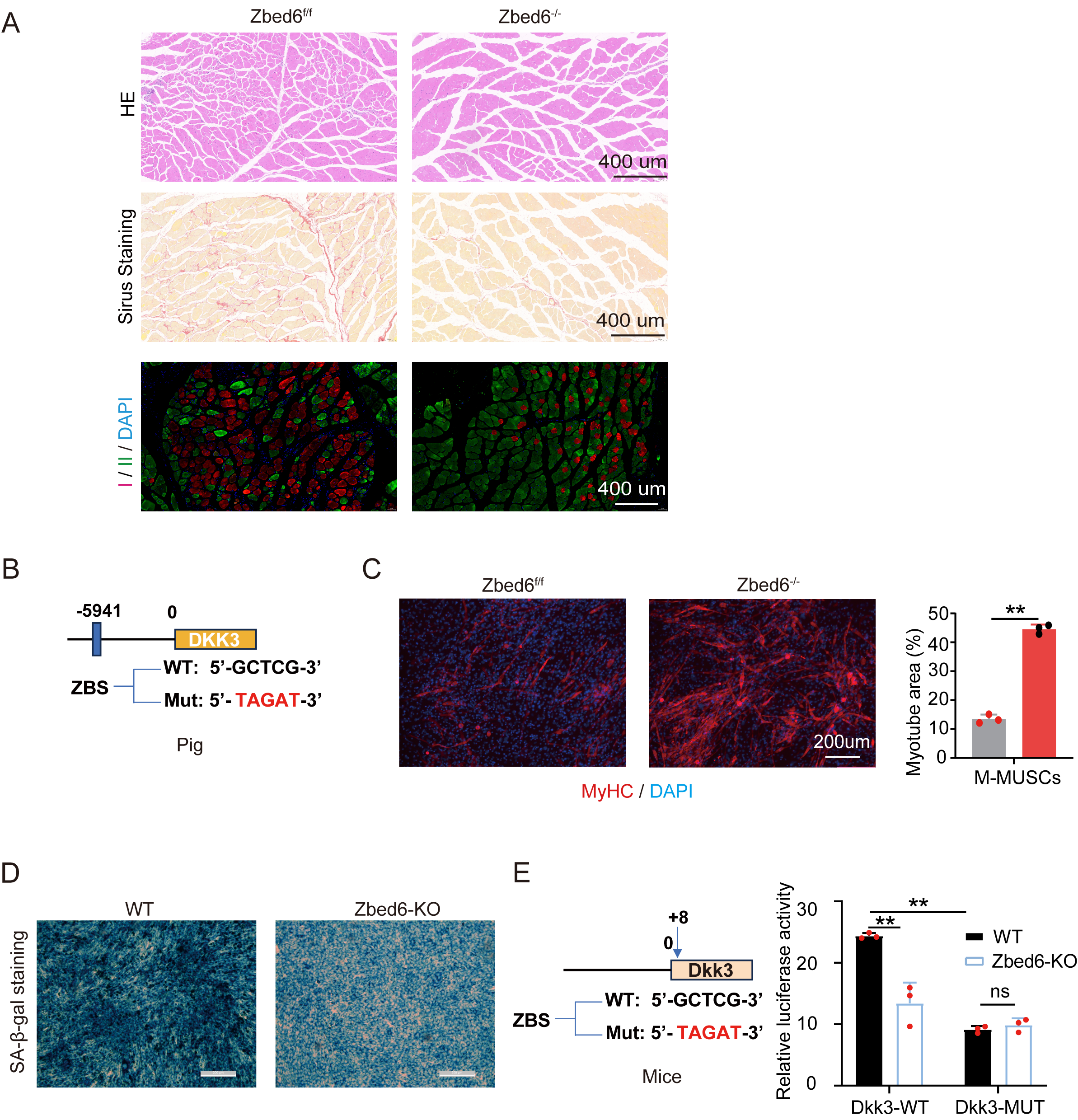

Supplement: Supplementary file 3 — Figure S3. Zbed6 controls Dkk3 transcription in ageing‐induced muscle atrophy. (A) Representative H&E staining (top), Sirius (middle) and fibre‐type staining (bottom) of histological cross sections in GM tissues of Zbed6−/− and controls of 18 months old mice. Myosin heavy chain type I and IIa (red), IIb (green) and DAPI (blue). Representative images are shown. Scale bar = 400 μm. n = 3. (B) The wild‐type and ZBS mutant sequences of pigs are indicated. (C) Representative images and quantification for myotubes area of primary skeletal muscle satellite cells from Zbed6−/− and controls. Red indicated myosin heavy chain (MyHC) immunofluorescent staining and DAPI (blue). Scale bars = 200 μm. n = 3. (D) Representative images of SA‐β‐gal staining in D‐gal‐induced C2C12 myoblasts of WT and Zbed6‐KO cells. Scale bars, 200 μm. (E) The wild‐type and ZBS mutant sequences of mice are indicated. Luciferase analysis showing the effects of WT and Zbed6‐KO C2C12 cells on mice wild‐type Dkk3‐ZBS luciferase (ZBS‐LUC) or mutant Dkk3‐ZBS luciferase (mZBS‐LUC). Data are expressed as mean ± SEM; *p < 0.05, **p < 0.01. [file JCSM-16-e13829-s006.tif]

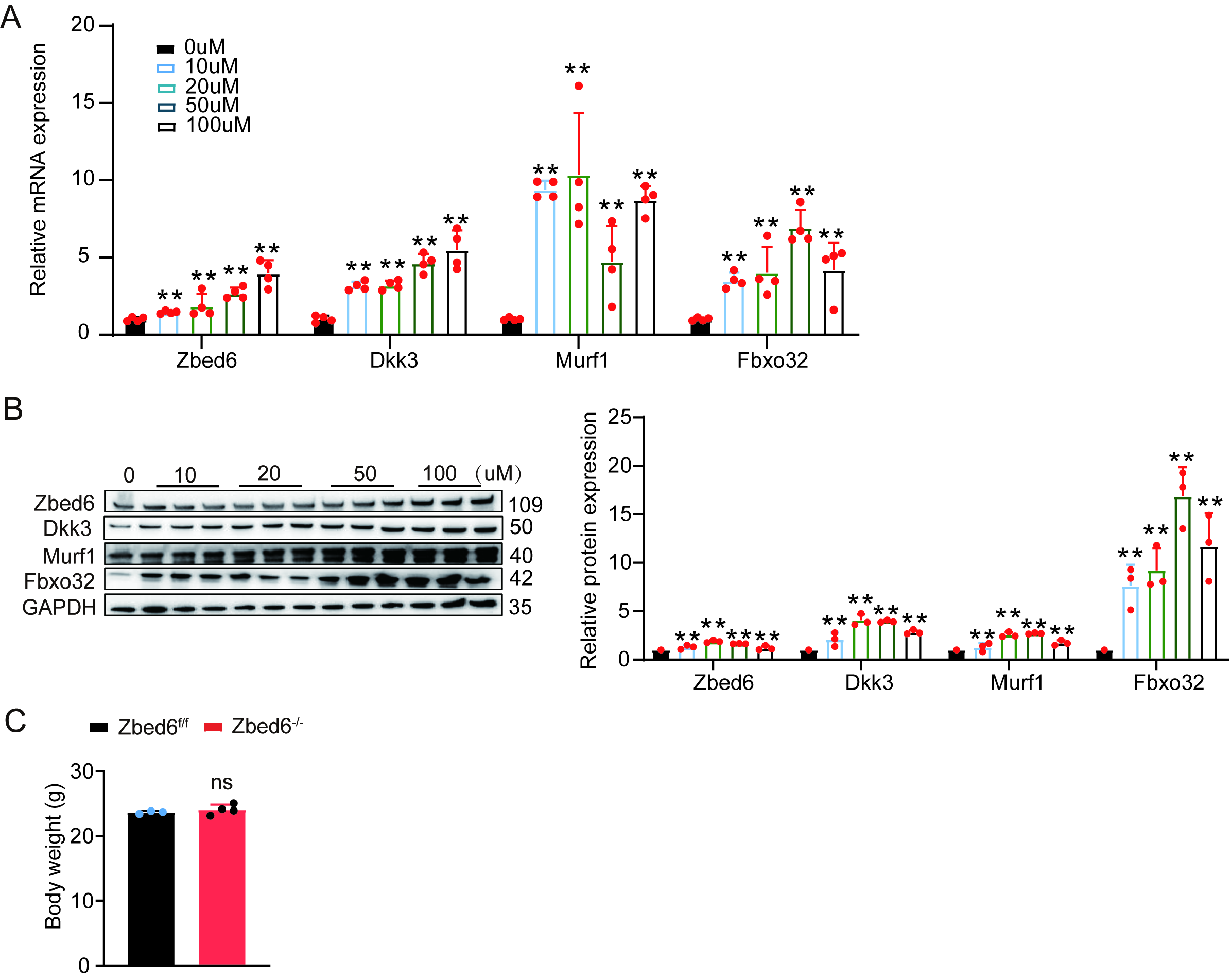

Supplement: Supplementary file 4 — Figure S4. Zbed6 depletion protects dexamethasone‐induced myotubes and muscles atrophy. (A) Zbed6, Dkk3, Fbxo32 and Murf1 mRNA expression level in Dex‐induced atrophy model of C2C12 myotubes (C(Dex) = 0, 10, 20, 50, 100 μM). (B) Representative western blotting and quantification of Zbed6, Dkk3, Fbxo32 and Murf1 in Dex‐induced atrophy model of C2C12 myotubes. (C) The body weight of Zbed6−/− and controls (3‐month‐old) for injected with or without 15 mg/kg/day of Dex for 10 days. GAPDH served as internal control. Data are expressed as mean ± SEM; *p < 0.05, **p < 0.01. [file JCSM-16-e13829-s002.tif]

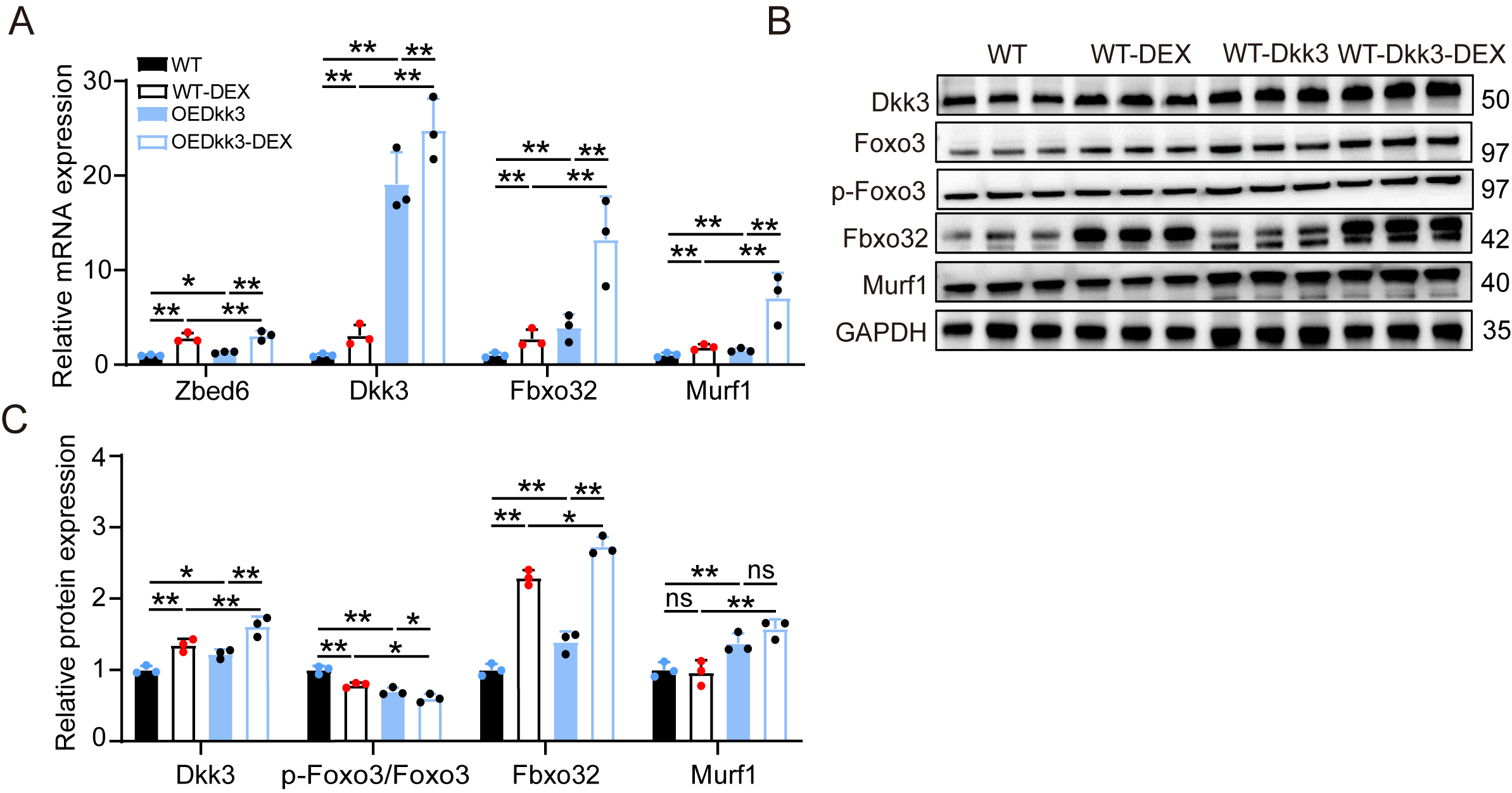

Supplement: Supplementary file 5 — Figure S5. Dkk3 mediates Dex‐induced muscle atrophy regulated by Zbed6. (A) Zbed6, Dkk3, Fbxo32 and Murf1 mRNA expression level of WT myotubes with overexpression Dkk3. Representative western blotting (B) and quantification (C) of Dkk3, Fbxo32, Murf1, phosphorylated protein levels of FoxO3 and total FoxO3 of WT myotubes with overexpression Dkk3. n = 3. GAPDH served as internal control. Data are expressed as mean ± SEM; *p < 0.05, **p < 0.01. [file JCSM-16-e13829-s007.tif]

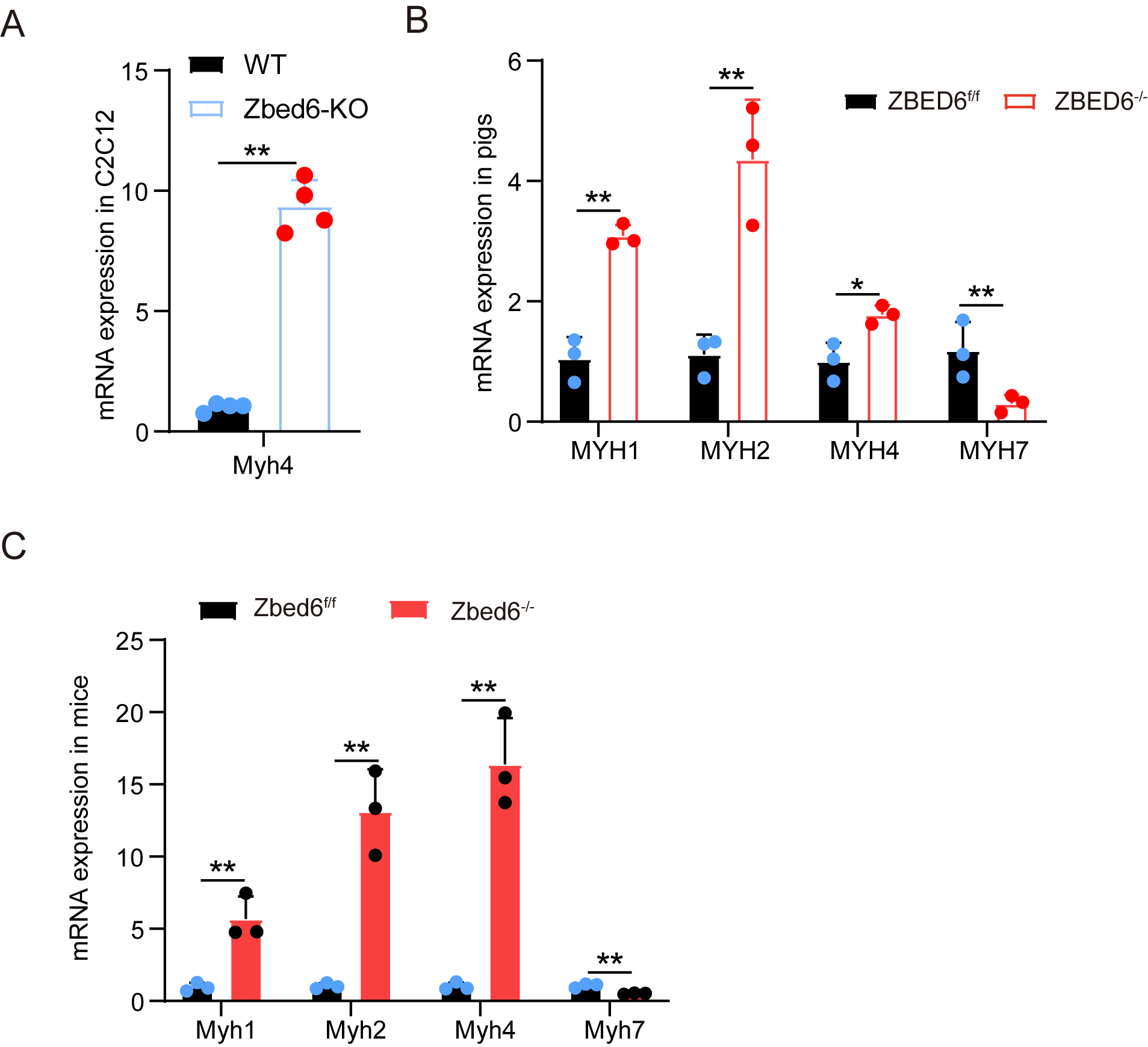

Supplement: Supplementary file 6 — Figure S6. The mRNA expression of Myh4 in pigs and mice. (A) Myh4 mRNA expression level of WT and Zbed6‐KO C2C12 myotubes. n = 4. (B) MYH1, MYH2, MYH4 and MYH7 mRNA expression level of wild‐type and ZBED6‐KO Bama pigs (8‐month‐old). n = 3. (C) Myh1, Myh2, Myh4 and Myh7 mRNA expression level of Zbed6f/f and Zbed6−/− mice (18‐month‐old). n = 3. GAPDH served as internal control. Data are expressed as mean ± SEM; *p < 0.05, **p < 0.01. [file JCSM-16-e13829-s001.tif]
