# Supplementary material for: ZBED6 Knockout Prevents Ageing‐ and Dexamethasone‐Induced Muscle Atrophy via Dkk3 in Pig and Mice
Source: J Cachexia Sarcopenia Muscle. 2025 Jun 4;16(3):e13829. doi: 10.1002/jcsm.13829 (PMC12134784; doi:10.1002/jcsm.13829)
Supplement: Supplementary file 8 — Data S1. Supporting Information [file JCSM-16-e13829-s008.doc]

**Methods**

***The dexamethasone model***

Intraperitoneal injections of Dex were administered at a dose of 15 mg/kg/day to 3-month-old male mice for 10 days to induce skeletal muscle atrophy.

***AAV injection***

Recombinant adeno-associated virus (AAV)-myo2A-ZBED6, AAV-myo2A-Dkk3, and AAV-myo2A-GFP vectors were constructed and purified by Cyagen. The AAV vectors were injected into the tibialis anterior (TA) muscle of 3-month-old male C57BL/6J mice. A total of 2×10^11^ genome copies of AAV-Zbed6 or AAV-Dkk3 were injected into the TA muscle on one side, while AAV-GFP was injected into the contralateral TA muscle of the same mice. The TA muscles were harvested after 30 days of injection.

***Immunoblotting analysis***

For the immunoblotting studies, proteins were extracted from the cells and muscle tissues using RIPA buffer. The cellular proteins were extracted as follows: the culture medium was initially removed, following which the cells were washed twice with phosphate-buffered saline (PBS). Then 150 μL of RIPA buffer was added to each well of a 12-well plate, and subsequently incubated on ice for 30 min to ensure complete lysis. The obtained lysates were centrifuged at 12,000 × g for 10 min at 4°C, and the supernatant was collected as the cellular protein extract. Additionally, 100 mg of muscle tissue was homogenized in 400 μL of RIPA buffer using a mechanical tissue homogenizer to extract tissue proteins. The homogenate was incubated on ice for 30 min and centrifuged at 12,000 × g for 10 min at 4°C, following which the supernatant was collected as the tissue protein extract. The protein lysates were subsequently quantified using a BCA protein assay kit (Invitrogen, USA), separated by SDS-PAGE (20 µg of protein per well), and transferred to a 0.45 µm PVDF membrane using a transfer system (Millipore, USA). Immunoblotting was subsequently performed using the following primary antibodies: anti-ZBED6 (1:1,000; HPA068807, Atlas Antibodies, Sweden), anti-Dkk3 (1:1,000; 10365-1-AP, Proteintech, China), anti-MYHC (1:400; MF-20, DSHB, USA), anti-TRIM63 (1:2,000; ab183094, Abcam, UK), anti-Fbxo32 (1:2000; ab168372, Abcam, UK), anti-FoxO3A (1:2,500; ab109629, Abcam, UK), anti-FoxO3A (phospho S253) (1:2,500; ab154786, Abcam, UK), anti-DYKDDDDK (1:1,000; 14793S, CST, DE), and anti-GAPDH (1:50,000; 60004-1-Ig, Proteintech, China) antibodies, The HRP-conjugated secondary antibodies used for immunoblotting included goat anti-mouse (1:5,000; SA00001-1; Proteintech, China) and goat anti-rabbit (1:5,000; SA00001-2; Proteintech, China) antibodies.

***Immunofluorescence staining and histology***

The frozen muscle tissue sections and cells were fixed in 4% paraformaldehyde for 30 min on ice, and permeabilised with 0.1% Triton X-100 for 10 min. The samples were then blocked by incubating with 10% goat serum for 1 h at room temperature, followed by overnight incubation with the anti-MyHC (1:40, MF20; DSHB, USA), anti-MYH1 (1:100; sc-32732, Santa Cruz, USA), anti-MYH7 (1:100; sc-53090, Santa Cruz), or anti-laminin (1:200, ab11575, Abcam, UK) primary antibodies at 4°C. The samples were then incubated with the HRP-conjugated secondary antibodies (Invitrogen), subjected to nuclear staining with DAPI, and finally mounted on slides.

Images of the H&E- and Sirius-stained sections were captured using a laser confocal microscope (TCS-SP8, Leica), while fluorescence imaging was performed using a fluorescent inverted microscope (DMI6000B, Leica) equipped with an objective of 10× magnification. The different fibre types and their CSAs were determined using the ImageJ software. The CSA of the muscle fibres was measured in two steps. First, the scale was set using the Line Tool to draw a straight line along the scale bar. The scale was subsequently defined from the “Analyze” menu, and the actual length and unit of the line was set to calibrate the image scale. Second, the CSA was measured using the Polygon Tool to manually outline the contours of the muscle fibres. The CSA of the selected muscle fibres was finally determined from the “Measure” option under the Analyze menu.

The diameter of the myotubes was subsequently measured as described hereafter. In the first step, the image was opened and the scale was set. The Line Tool was then used to draw a line along the scale bar, following which the actual length and unit of the line were defined from the "Set Scale" option under the "Analyze" menu. In the second step, the diameter of the myotubes was measured from the Line Tool menu. The diameter of at least six myotubes was measured in each field of view, following which the average diameter was calculated. The measurements from three different fields of view were used to generate the final plot. The area of the myotubes was measured as previously described [18].

***ChIP-Seq and ChIP-PCR***

ChIP assays were performed as previously described [15], and ChIP-PCR was conducted as previously reported using primers enlisted in Table S1 of the Supporting Information section.

***RNA-seq analysis***

RNA‐seq libraries were constructed from the GM tissues of three WT and ZBED6-KO pigs. Transcriptome analysis was performed using 150 cycles of paired-end sequencing on an Illumina HiSeq 2500 platform (Berry Genomics Company, Beijing). The reference pig genome (version Sscrofa11.1) was retrieved from the Ensembl database (ftp://ftp.ensembl.org/pub/release-101/fasta/sus_scrofa/dna/Sus_scrofa.Sscrofa11.1.dna.toplevel.fa.gz). The cleaned sequence reads were aligned to this reference genome using HISAT2 (v2.1.0) with default parameters (hisat2 -x index -1 RNA-data_1.fq -2 RNA-data_2.fq -S RNA-data.sam). The gene expression levels were quantified using the featureCounts program, while differential gene expression between the WT and ZBED6-KO groups was analysed using the DEseq2 package of R. The genes with q values < 0.05 and a fold change > 2 were classified as differentially expressed genes (DEGs).

***Luciferase reporter assay***

The promoter region of the gene encoding Dkk3 was amplified from WT pigs and mice, and subsequently cloned and inserted into the PGL4.10 basic luciferase reporter vector. HEK293T cells cultured in 12-well plates were transiently transfected with PGL4.10-Dkk3-WT, PGL4.10-Dkk3-MUT, or PGL4.10 vectors (1,600 ng/well) along with PRL-TK vector (160 ng/well). The cells were subsequently cultured in DMEM supplemented with 10% serum, and increasing concentrations of the ADV-control and ADV-ZBED6 vectors. The activity of luciferase was measured after 48 h of transfection, using the Dual Luciferase Reporter Assay Kit (E1910, Promega, USA). Six independent reactions were performed for each experimental condition, and the experiments were performed in triplicate.

***RNA and RT-qPCR***

Total RNA was extracted from porcine and murine muscles and cultured cells using TRIzol Reagent (R711, Vazyme, China). The total RNA was reverse transcribed to cDNA using the HiScript III RT SuperMix for qPCR (+gDNA wiper) (R323-01, Vazyme, China) and qPCR was performed in triplicate using the Taq Pro Universal SYBR qPCR Master Mix (R712-02, Vazyme, China). The results were analysed using the software provided with the Q7 Optical System (ABI, USA). The primers used for RT-qPCR analysis are enlisted in Table S1.

***Data availability statemen*t**

The datasets generated and analysed in this study are available in the NCBI Sequence Read Archive (SRA) database. The RNA-seq reads were deposited in the SRA database (https://www.ncbi.nlm.nih.gov/sra) under the BioProject accession ID: PRJNA663759.

***Statistical analyses***

Statistical significance was assessed in this study using two methods, as described hereafter. The data from multiple groups were initially compared by one-way or two-way analysis of variance, in conjunction with Tukey’s multiple comparison tests at 5% level of significance, to determine the differences in the mean values. A two-sided Student’s t-test was also employed to identify statistically significant differences between two groups. The data were graphically represented using the GraphPad Prism software, version 8, with error bars indicating the standard error of the mean. The experiment was conducted with three independent replicates, and statistical significance was considered at P < 0.05; **P* < 0.05 and ***P* < 0.01.
